# Supplementary material for: The electron–proton bottleneck of photosynthetic oxygen evolution
Source: Nature. 2023 May 3;617(7961):623–8. doi: 10.1038/s41586-023-06008-5 (PMC10191853; doi:10.1038/s41586-023-06008-5)
Supplement: Supplementary file 1 — Supplementary information on the methods and a supplementary discussion for both the step-scan FTIR experiments and the computational chemistry. Moreover, a scheme detailing Fig. 1c is presented. The file includes supplementary text sections, 22 supplementary figures and 3 supplementary tables. An internal table of contents shows the order of the supplementary elements. [file 41586_2023_6008_MOESM1_ESM.pdf]

nature portfolio

https://doi.org/10.1038/s41586-023-06008-5

Supplementary information

The electron–proton bottleneck of photosynthetic oxygen evolution

In the format provided by the authors and unedited

SUPPLEMENTARY INFORMATION

The electron-proton bottleneck of photosynthetic oxygen evolution

Paul Greife[1#], Matthias Schönborn[1#], Matteo Capone[2,3#], Ricardo Assunção[1], Daniele Narzi[3], Leonardo Guidoni[3]*, Holger Dau[1]*

[1]Dept. of Physics, Freie Univ. Berlin, Germany; [2]Dept. of Information Engineering, Computer Science and Mathematics, Univ. of L'Aquila, Italy; [3]Dept. of Physical and Chemical Sciences, Univ. of L'Aquila, Italy

[#]These three authors contributed equally.

*Corresponding authors: holger.dau@fu-berlin.de, leonardo.guidoni@univaq.it

Table of contents

	Pg.
**Supplementary Information on Figure 1c**	3
**Supplementary Fig. 1** Version of Figure 1c with H-bond distances, sidechain assignments, and numbering of atoms of the $Mn_4Ca(\mu\text{-}O)_5(H_xO)_5$ cluster.	3
**SI. Supplementary Information – Step-scan FTIR Experiment**	3
**SI.1** Experimental set-up for step-scan FTIR with automated sample-exchange system	3
**SI.2** Heat artefact correction	4
**Supplementary Fig. 2** Illustrative spectra and time courses on correction for laser-induced heating	5
**SI.3** Aging of the PSII samples for prolonged experimentation	6
**Supplementary Fig. 3** Stability of PSII function for up to 60 hours as judged from S-state difference spectra	6
**SI.4** Primary FTIR data analysis	6
**SI.5** Pure S-state transitions by deconvolution	7
**Supplementary Tab. 1** Flash-number dependence of S-state population	9
**Supplementary Fig. 4** Spectra and time courses illustrating S-state deconvolution	10
**SI.6** Correction for acceptor side contributions	11
**Supplementary Fig. 5** Correction for acceptor side contributions of the DAS	12
**SI.7** Simulation parameters for O2-evolution transients from polarography	13
**Supplementary Fig. 6** Complete sets of fit parameters in simulation of the O2-evolution transients	13
**SI.8** Time constants in simulation of S-state transitions	14
**Supplementary Tab. 2** Fit parameters for the transients in Figure 1 and Extended Data Fig. 2	14

**Supplementary Tab. 3** Time-constant values for S-state cycle reactions from previous investigations	15
**SI.9** Spectrokinetic signatures of carboxylate sidechain deprotonation and potentially overlapping Tyr_Z contributions	16
**Supplementary Fig. 7.** Population-associated spectra (1700-1800 cm^{-1}) for assessment of Tyr_Z contributions	16
**Supplementary Fig. 8.** IR transients at prominent Tyr_Z^{ox}/Tyr_Z wavenumbers	17
**Supplementary Fig. 9.** S-state IR difference spectra from 1700-1800 cm^{-1}.	18
**SI.10** Comparison of DAS for data sets collected in deuterated water	19
**Supplementary Fig. 10.** Difference spectra for PSII in H_2O and D_2O at 14 ms	21
**Supplementary Fig. 11.** DAS in H_2O and D_2O.	22
**Supplementary Fig. 12.** Comparison of DAS and time courses in H_2O and D_2O	23
**SI.11** Deprotonation in S_3->S_3' transition assigned to Glu65—Glu312 dyad	24
**Supplementary Fig. 13.** DAS of Figure 2 shown for an extended spectral range	25
**SII. Supplementary Discussion – Computational Chemistry**	27
**SII.1** Exploring protonation states of carboxylate groups in the S3 – Glu61 (D61)	27
**Supplementary Fig. 14** Geometry optimization analysis of an Asp61 protonation	27
**SII.2** Exploring protonation states of carboxylate groups in the S3 – Glu65 (E65)	28
**Supplementary Fig. 15** Electrostatic stabilization favoring deprotonated E65	29
**Supplementary Fig. 16** Comparison between X-ray structure and computationally optimized structures	29
**Supplementary Tab. 4** Interaction distances of the E312 hydrogen bond network	30
**SII.3** Possible role of Lys317	30
**Supplementary Fig. 17** RMSD of the protein backbone along the MM simulations	31
**SII.4** The minimum energy path of proton-coupled electron transfer (i→ii) and peroxo-bond formation (ii→iii)	31
**Supplementary Fig. 18** Complete QM region used in the QM and QMMM calculations	31
**Supplementary Fig. 19** Schematic representation of nudged elastic band (NEB) method employed in the present study in the Minimum Energy Path (MEP) calculations	32
**Supplementary Fig. 20** The 14 optimized structures corresponding to the calculated Minimum Energy Path	33
**SII.6** Comparison between present MEP calculations with previous literature	34
**SII.7** Consideration about Proton Coupled Electron Transfer (PCET) in S_3->S_0 transition	35
**SII.8** Applicability of transition state theory for proton-coupled electron transfer	36
**Supplementary Fig. 21** Shortest distances between the sidechain of Tyr161 (Tyr_Z) and the first-sphere ligand atoms	37
**SII.9** Entropic activation-energy contribution in the slow phase of the S_3 to S_0 transition	38
**Supplementary Fig. 22** Hydrogen bond network distances around Tyr_Z and O6	40
**References**	42

Supplementary information on Figure 1c

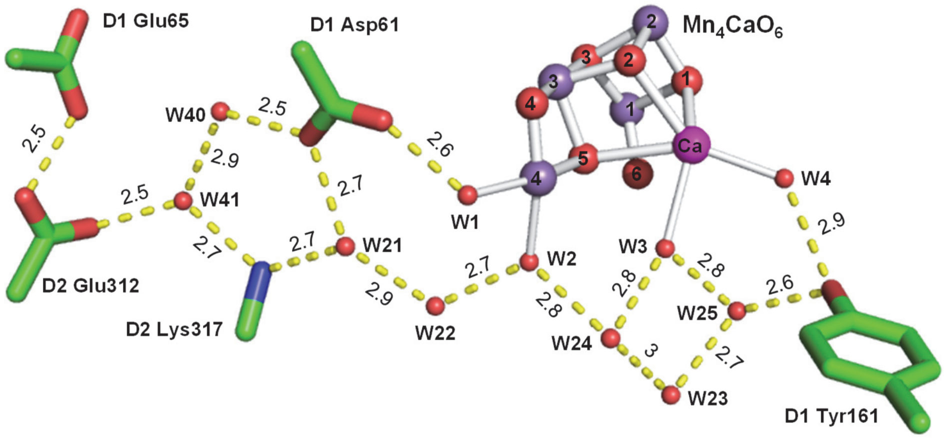

Supplementary Fig. 1. Version of Figure 1c with H-bond distances, sidechain assignments, and numbering of the atoms of the $Mn_4Ca(\mu\text{-}O)_5(H_xO)_5$ cluster. In comparison to Figure 1c, the image is rotated clockwise by 90°. Indicated H-bond distances in Å. The residues are part of three subunits of the photosystem II protein complex called D1 (PsbA gene product), D2 (PsbB), and CP43 (PsbC). The water molecules are numbered following Hussein et al (2021). [21] Atoms of the metal cluster are denoted as Mn1 to Mn4, O1 to O6, and W1 to W4. The atom coordinates are from ref. 25 and relate to the S_3-state; a further light-flash initiates the sequence of events which leads to formation of an O-O bond between O5 and O6.

SI. Supplementary Information – Step-scan FTIR Experiment

SI.1 Experimental set-up for step-scan FTIR with automated sample-exchange system

The experimental setup consists of a commercial FTIR spectrometer (Bruker, Vertex 70) modified with an extended sample chamber and temperature control device, as illustrated in the top panel of Extended Data Fig.1. The beam path of the spectrometer has been modified to include two IR filters (Quantum design), a low-pass with 73% transmission at 1100 cm^{-1} and a high-pass with 80% transmission at 1800 cm^{-1}, separating the sample chamber from the interferometer and detector sections, respectively. These bandwidth limiting filters facilitates undersampling of the interferometer positions resulting in a reduced number of 334 positions for a two-sided interferogram. The two IR filters also act as visible-light filters blocking the visible-light excitation laser flash.

Two ellipsoidal focusing mirrors focus the IR beam onto a sample spot, resulting in a focused IR-beam with a diameter of 1.6 mm. The diameter of the spot illuminated by nanosecond flashes of the visible-light laser exceeded the IR-spot diameter. The visible-light laser was a pulsed frequency-doubled NdYag laser (532 nm, ca. 5 ns FWHM). In preliminary experiments, the pulse energy was adjusted for full saturation of the photosynthetic reactions. Timing of data acquisition and excitation laser was handled by a multi-channel pulse generator; for a timing diagram, see Extended Data Fig. 1. Self-written software (in C#) orchestrates the movement of the sample exchanger, refilling of the nitrogen

Dewar to control the temperature, triggering of the pulse generator, and sends commands to the spectrometer through the HTML interface of the Vertex system.

SI.2 Heat artefact correction

All experimental data was processed and analyzed using Python, with standard Python toolboxes and scripts adapted for implementation of the equations and data analysis procedures described in the Methods section of the article and in the Supplementary Material, in the following sections.

Correction for heat-induced changes in spectra due to temperature fluctuations caused by absorption of the exciting laser-flash light is essential for proper evaluation and interpretation of time-resolved FTIR data. Supplementary Fig. 2 illustrates the effect of the phenomenon, with a significant influence within 100 µs after the laser flash. Therefore, we approached a series of experiments for determination of the artifact contribution and subsequent correction of the step-scan data set that has been collected as describe above. Briefly, a PSII sample was prepared for FTIR measurements without variation of the protocol described above, but without any artificial electron acceptor. Thirty pre-flashes were applied to each sample spot to completely reduce the native quinone electron acceptors, thereby ensuring that subsequent flashes can no longer induce light-driven charge separation by the PSII particles. At each interferogram mirror position, three excitation flashes with about 2.4 times the laser-flash energy used for data collection on functional PSII were applied at 15 Hz laser-flash frequency. In repetitions of these experiments, we observed undesired signal contributions of variable strength, possibly assignable to formation of carotenoid triplet states. We corrected for these signal contributions by difference calculations, exploiting that the undesired signals are observable with reproducible spectral shape but variable amplitude. A resulting spectrum that represents the thermal artifact at 9 µs after application of the excitation flash is shown in Supplementary Fig. 2 a, and made available in ref. 54. We verified that (i) the time evolution of the thermal artifact is uniform across its spectrum and (ii) corresponds exactly to the time course of a thermal artefact signal visible with equal strength in all interferograms signals. Then the time evolution was extracted as the average of the interferograms, which is shown in Supplementary Fig. 2 b. This extracted, time-resolved thermal artifact was then fitted to the regular step-scan data set by minimizing the sum of least squares simultaneously. The spectral and temporal results are shown in Supplementary Fig. 2 a, c-f. The effects range from minimal influence in the carboxylate stretching region to a complete reversal of the trend in other regions, potentially casting doubt on the source of these early kinetics. However, a closer examination of the results shows a clear four-period behavior with different early kinetics. This is particularly clear at 1571 cm^{-1}, where the first and fifth flashes are stagnant (Supplementary Fig. 2 f) and the third and seventh flashes (Supplementary Fig. 2 e) show an increase.

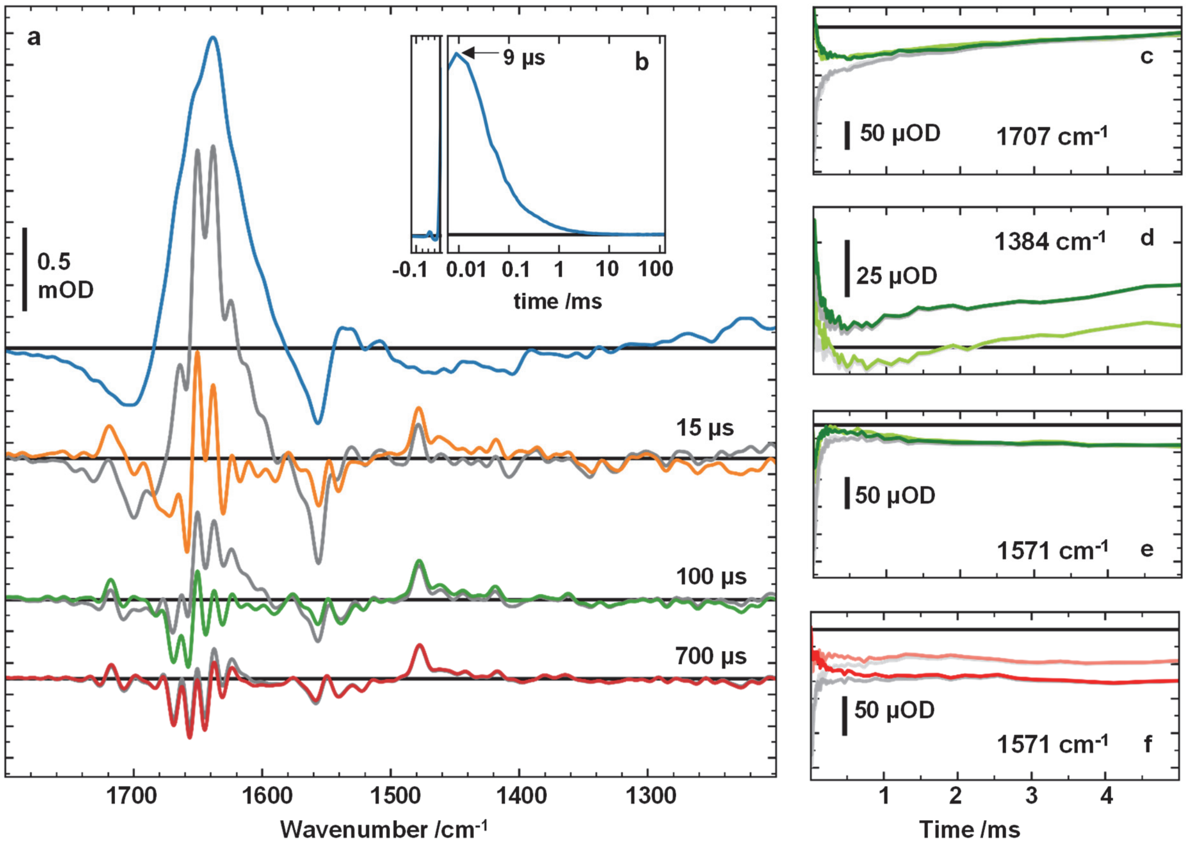

Supplementary Fig. 2. Illustrative spectra and time courses on correction for laser-induced heating (heat artifact, see above text). In **a**, blue, scaled heat artefact spectrum; grey, uncorrected third flash spectra at various times; colored, heat artefact corrected third flash spectra. In **b**, heat artefact transient recorded at center-burst position of the interferogram, note the linear time axis before the flash, and the logarithmic time axis after. In **c-e**, third-flash (dark green) and seventh-flash (light green) corrected transients and (grey lines) uncorrected transients. In f, first-flash (dark red) and fifth-flash (light red) corrected transients and (grey lines) uncorrected transients.

SI.3 Aging of the PSII samples for prolonged experimentation

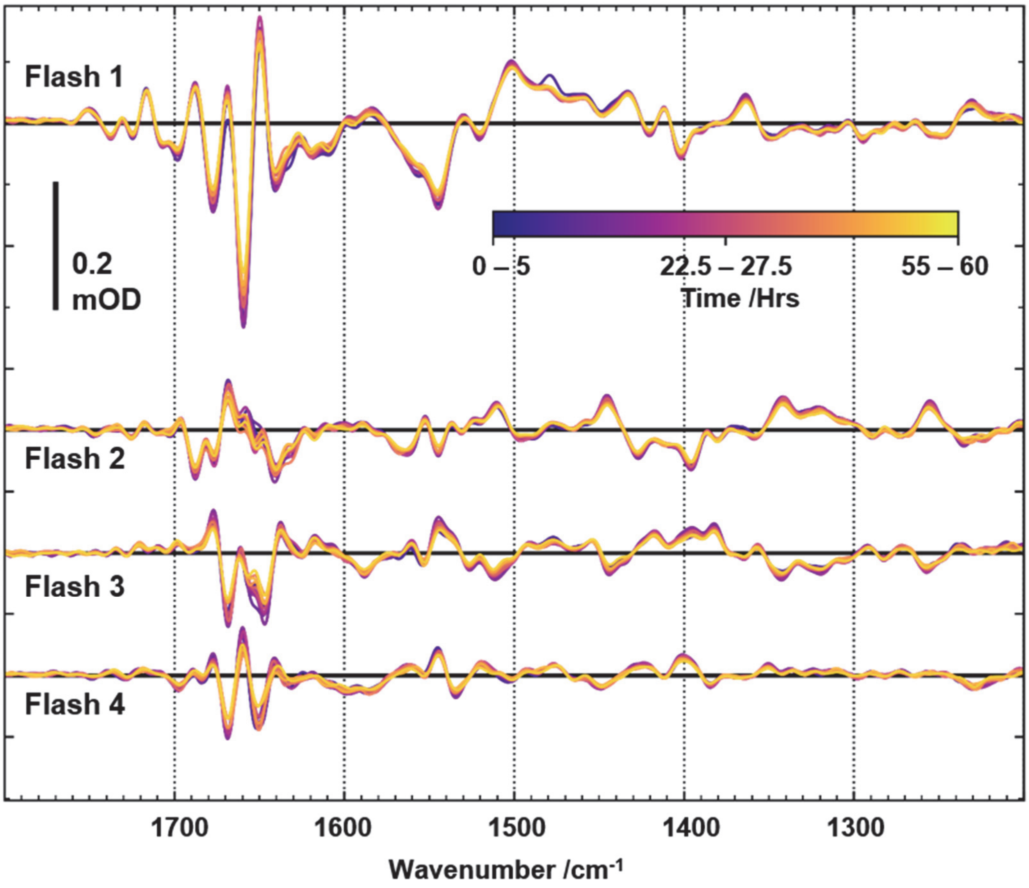

Supplementary Fig. 3. Stability of PSII function for up to 60 hours as judged from S-state difference spectra. A rapid-scan measurement recorded with a spectral resolution of 10 cm^{-1} (at 10°C) over several days, averaged in batches of 5 hours. Each batch contains roughly 1000 flash sequences of 10 excitation flashes applied with a flash spacing of 700 ms. The remaining measurement parameters are identical to those described in the Materials and Methods section. The shown data demonstrates that spectral differences between the different batches collected over a time period of 60 hours are negligible.

SI.4 Primary FTIR data analysis

Step-scan transients were normalized to rapid-scan interferogram counterparts, to account for variations in sample thickness. Prior to averaging, a Forman phase correction was applied to stitched interferograms. [85] The phase correction uses a truncated interferogram to calculate the phase spectrum and correct for mismatches in the interferogram between data sets. Here, of the original 334 mirror positions only the central 320 are used to calculate the phase spectrum. To calculate the location of the center burst, the positions of the minimum and maximum values were averaged and rounded. We observed oscillatory noise contributions of the IR transients resulting from small oscillations in the center-burst position of the interferometer caused by acoustically excited small-amplitude oscillation of the interferometer mirror position. These were effectively minimized by setting the absorption change for the six mirror positions closest to the center burst to zero. We

verified by simulation that, as expected, there was no effect on the time-resolved spectra itself aside from the (desirable) removal of broad background features.

To reduce data set size, the transients (time courses) at the respective wavenumber were averaged such that the number of data points per decade was 24, with equidistant position of the data point on a logarithmic time axis (logarithmic averaging; for 69 µs before and 153 µs after the laser flash, the logarithmic averaging was not applied). Data is made available at this stage of the analysis in ref. 54 with the centerburst still intact. Finally, the real component of the Fast Fourier Transform (FFT) was computed, and the infrared absorption calculated.

SI.5 Pure S-state transitions by deconvolution

By application of 10 light flashes in our FTIR experiment, the PSII cycle up to 2.5 times through the four semi-stable intermediates of the S-state reaction cycle. The cycling does not proceed fully synchronized because of 'miss events' that result in less-than-unity efficiency (Φ) of advancement in the S-state cycle (Φ = 100% - *m*, where *m* is the 'miss factor'). We developed a deconvolution procedure that takes into account all the IR transients collected starting from the 2nd flash up to the 10th flash. By omitting the first flash from the analysis, we ensured that events taking place exclusively on the first flash do not affect the results of our analysis, specifically the stable (for several seconds) formation of reduced primary quinone acceptor (Q_A^-) in photosystems without a functional Q_B site.

Let $P_{i,j}$ be the matrix representing the populations over the course of the experiment, where *i = 0, 1, 2, .. i* is the flash number and *j = 1, 2, 3, 0* indicates the S-states. Before the first flash, it is assumed that the initial population is shared between the S_1 state and S_0 state, that is, $P_0 = (1-S_0,0,0,S_0)$. Subsequent flash populations can be calculated using the propagation matrix *M*(*m*) with miss factor *m:*

$$P_{i+1}(m) = M(m) \cdot P_i = \begin{pmatrix} m & 0 & 0 & 1-m \\ 1-m & m & 0 & 0 \\ 0 & 1-m & m & 0 \\ 0 & 0 & 1-m & m \end{pmatrix} \cdot P_i = (M)^{i+1} \cdot P_0$$

The above population vector contains the distribution of the photosystems across each of the four S-states after the ith flash. The signal registered at the detector, *R*, differs from the population propagation because only those photosystems that successfully transition are assumed to contribute. This means that a modified version of the matrix *M*, representing only those centers participating in a transition, must be applied to the vector P_i to calculate the contribution of each S-state to the signal induced by the i+1 flash.

$$R_{i+1}(m) = \begin{pmatrix} 0 & 0 & 0 & 1-m \\ 1-m & 0 & 0 & 0 \\ 0 & 1-m & 0 & 0 \\ 0 & 0 & 1-m & 0 \end{pmatrix} \cdot P_i$$

Note that this participation matrix is simply the propagation matrix M(m) with zeros along the diagonal, as it is assumed that centers experiencing a 'miss' do not contribute to the signal.

The flash dependent amplitudes $A_{i,\nu}$, where ν is the wavenumber, can be derived from the S-state eigenvalues α_j and the signal matrix $R_{i,j}$.

$$A_{i,\nu} = R_{i,j} \cdot \alpha_\nu^j$$

To solve for α, a matrix inversion must be applied to a symmetric matrix

$$R^T \cdot A = R^T \cdot R \cdot \alpha$$

$$\alpha = (R^T \cdot R)^{-1} \cdot R^T \cdot A$$

The element ($R^T \cdot R$) is degenerate so the pseudo-inverse must be approximated using a least-squares solver. The miss factor m found by calculating the difference between A_ν and the simulated $A_\nu(m)$ and minimizing the sum of their squares:

$$\left(\sum_{i,\nu} A_{i,\nu} - A'_{i,\nu}(m)\right)^2 = \left(\sum_{i,\nu} A_{i,\nu} - R \cdot (R^T \cdot R)^{-1} \cdot R^T \cdot A_{i,\nu}\right)^2 \rightarrow 0$$

The above deconvolution approach in implemented and made available in ref. 55.

In addition, extended dark-adaptation periods cannot ensure that all PSII are in the dark-stable S_1 state. Minor contributions of further S-states (specifically the S_0 state) were considered, as described in the following. The long-time dark-stable S-States are S_1 and S_0; as such it was assumed that the initial population in other states was negligible. In the S_1 -> S_2, S_2 -> S_3 and S_0 -> S_1 transition, the millisecond phase corresponds to the redox reaction between Q_A and PPBQ. As the two electron quinone chemistry repeats itself after a two S-state transitions it was assumed that the corresponding decay associated spectra, $\alpha_\tau^{i\text{->}i+1}$ of the S_2->S_3 transitions should look very similar to its parallel in the S_0->S_1 transition.

$$\alpha_{1.7ms}^{S2\rightarrow S3} \approx \alpha_{1.7ms}^{S0\rightarrow S1}$$

During the S_3->S_0 transition, this phase happens to be concurrent with the dominating oxygen-evolution phase. As such, it will be the most different of the four transitions, with unique O-O bond formation features alongside the quinone features. These similarities and differences can be observed in Supplementary Fig. 4 f.

$$\alpha_{2.7ms}^{S3\rightarrow S0} = \alpha_Q^{S3\rightarrow S0} + \alpha_{O2}^{S3\rightarrow S0}$$

The components of the S_3->S_0 DAS corresponding to quinone chemistry should be also very similar to that of the S_1->S_2 transition.

$$\alpha_{Q}^{S3\rightarrow S0} \approx \alpha_{1.7ms}^{S1\rightarrow S2}$$

Exploiting this pattern and the unique signals seen in the S_3->S_0 transition the fraction of centers starting in the S0 state was approximated by minimizing the ratio of the difference between the millisecond DAS the S_2 -> S_3 and S_0 -> S_1 transitions while maximizing the difference between the S_1 -> S_2 and S_3 -> S_0 transitions.

$$\min\left(\frac{\sum_{\nu}\left|\alpha_{1.7ms}^{S2\rightarrow S3} - \alpha_{1.7ms}^{S0\rightarrow S1}\right|}{\sum_{\nu}\left|\alpha_{2.7ms}^{S3\rightarrow S0} - \alpha_{1.7ms}^{S1\rightarrow S2}\right|}\right)$$

Thereby the parameters for S-state deconvolution were obtained. The deconvolution resulted in corrected transients, which correspond to 'pure' S-state transitions. For deconvolution, flashes 2 through 10 were used with a miss factor of 13.8% and initial S-state population of 87.7% S_1/12.3% S_0.

	S_1	S_2	S_3	S_0
Dark Adapted	87.7	0	0	12.3
Flash 1	22.7	75.6	0	1.7
Flash 2	4.6	30.0	65.2	0.2
Flash 3	0.8	8.1	34.9	56.2
Flash 4	48.6	1.8	11.8	37.8
Flash 5	39.3	42.1	3.2	15.4
Flash 6	18.7	39.7	36.7	4.9
Flash 7	6.8	21.6	39.3	32.4
Flash 8	28.8	8.8	24.0	38.3
Flash 9	37.0	26.1	10.9	26.0
Flash 10	27.5	35.5	24.0	13.0

Supplementary Tab. 1. Flash-number dependence of S-state population. Estimated S-state populations (in percent) of dark-adapted PSII and after the respective laser flash, for a best-fit miss factor of 13.8% (m = 0.138). The propagation of the S_0 and S_1 populations are plotted in Supplementary Fig. 4 i as the dashed magenta and red lines. We note that the eventually analyzed step-scan data corresponds to 'pure' S-state transitions, as obtained by the S-state deconvolution approach described above.

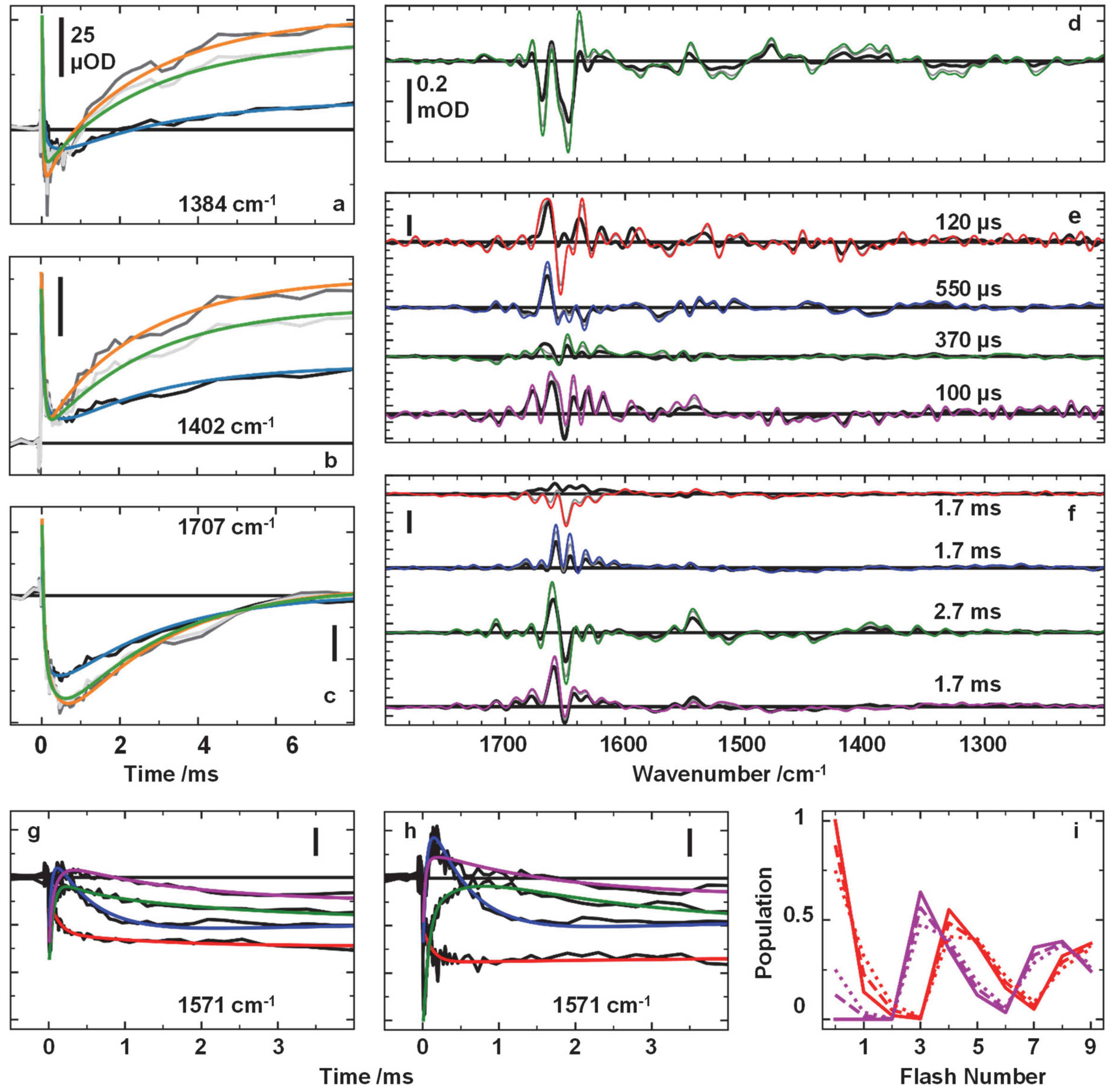

Supplementary Fig. 4. Spectra and time courses illustrating S-state deconvolution, that is, the correction for impure S-state populations as they result from imperfectly synchronized advancement in the S-state cycle.

a, b, c. Transients comparing the effect of the deconvolution on the S_3 -> S_0 (3rd flash) transition with different starting populations at various wavenumbers. Blue, 'raw' (as measured) 3rd flash transient; green, S_3 -> S_0 deconvoluted transient with 100% S_1 starting population; orange, S_3 -> S_0 deconvoluted transient with 87.7% S_1 and 12.3% S_0 starting populations.

d. S_3 -> S_0 Spectra at 130 ms after the flash. Black, 'raw' 3rd flash spectrum; grey, S_3 -> S_0 deconvoluted spectrum with 100% S_1 starting population; green, S_3 -> S_0 deconvoluted spectrum with 87.7% S_1 and 12.3% S_0 starting populations.

e. Decay-associated spectra corresponding to the ~100 μs time constants of each transition. Colored, DAS spectra with 87.7% S_1 and 12.3% S_0 starting population; grey, 100% S_1 deconvolution variants; black, 'raw' flash data fitted with the same time constants as found in the deconvoluted counterparts. In descending order, S_1 -> S_2 (red), S_2 -> S_3 (blue), S_3 -> S_0 (green), S_0 -> S_1 (magenta). Note the good agreement of the S_1 -> S_2 and the 1st flash spectra.

f. Decay-associated spectra corresponding to the 1-3 ms processes in each transition (color code as in **e**). Note the poor agreement of the $S_1 \rightarrow S_2$ and the 1st flash spectrum, which relates to specifics of the acceptor side processes visible on the 1st flash only. Therefore, exclusively flashes 2 through 10 were used for obtaining pure S-state spectra by deconvolution.

g. 'Raw' transients at 1571 cm^{-1}. In red, 1st flash; blue, 2nd flash; green, 3rd flash; magenta, 4th flash.

h. 87.7% S_1 and 12.3% S_0 deconvoluted transients at 1571 cm^{-1}. In red, $S_1 \rightarrow S_2$; blue, $S_2 \rightarrow S_3$; green, $S_3 \rightarrow S_0$; magenta, $S_0 \rightarrow S_1$.

i. simulation of the S_1 (red) and S_0 (magenta) state populations following each flash with different starting parameters. Solid line, 100% S_1 and 0% S_0, dashed line, 87.7% S_1 and 12.3% S_0, dotted line, 75% S_1 and 25% S_0.

SI.6 Correction for acceptor side contributions

Concomitant to the oxygen evolution step of the $S_3 \rightarrow S_0$ transition, there are acceptor side processes associated with quinone reactions. While relatively small, the acceptor side contributions complicate interpretation of the spectra. Unlike the period-of-four pattern in the flash-number dependence of the S-state cycle reactions, the acceptor side functions with a period of two. Features of the 3.1 ms phase of the $S_1 \rightarrow S_2$ transition should then represent the same changes as the process in the $S_3 \rightarrow S_0$ transition. Analogous to the simulations of the $S_3 \rightarrow S_0$ transition, the $S_1 \rightarrow S_2$ transition was simulated using a multi-exponential function. The decay associated spectra of the 3.1 ms phase was then subtracted, without scaling, from 2.5 ms DAS of the $S_3 \rightarrow S_0$ transition. The effect of this operation becomes clear in two key regions: (1) between 1460 cm^{-1} and 1500 cm^{-1} two features associated with the quinone redox reactions become significantly smaller; (2) bands above 1700 dipping below the zero-line are shifted up, clarifying their position and significance to the oxygen evolution step; see Supplementary Fig. 5. To test the validity of this approach a time resolved spectrum of the 3.1 ms phase of the $S_1 \rightarrow S_2$ transition was simulated and subtracted from the time resolved $S_3 \rightarrow S_0$ data. (Simulation parameters are summarized in Supplementary Tab. 2 in section SI.8).

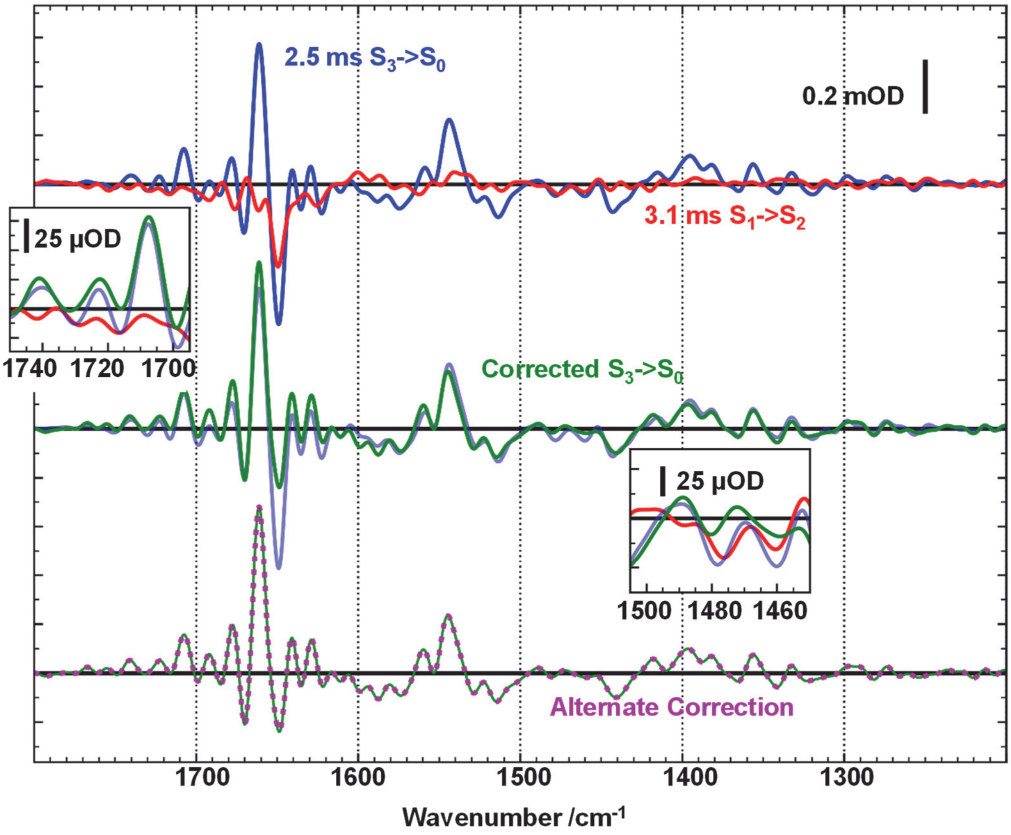

Supplementary Fig. 5. Correction for acceptor side contributions of the DAS of the oxygen-evolution step. Top: Comparison of the 2.5 ms / S_3 -> S_0 DAS (blue) and the 3.1 ms / S_1 -> S_2 DAS (red). Middle: Comparison of acceptor side corrected S_3 -> S_0 DAS (green) with the uncorrected DAS (blue). The correction is a simple subtraction of the S_1 -> S_2 DAS from the S_3 -> S_0 DAS. Of note is the region above 1700 cm^{-1} where spectral peaks move to the zero line and around 1470 cm^{-1} when features associated with the acceptor side disappear (see inserts). Bottom: Comparison of two correction methods. In green, the result of subtracting the acceptor side 3.1 ms / S_1 -> S_2 DAS from the 2.5 ms / S_3 -> S_0 DAS associated with the rate-determining step in O_2-formation. In red, an alternative where the 3.1 ms / S_1 -> S_2 DAS is used to simulate a transient and is then subtracted from the time-resolved S_3 -> S_0 data. Following this operation, the data is simulated with a time constant at 2.5 ms, representing the rate-determining step in O_2-formation. The two correction modes lead to a highly similar result.

SI.7 Simulation parameters for O_2-evolution transients from time-resolved polarography

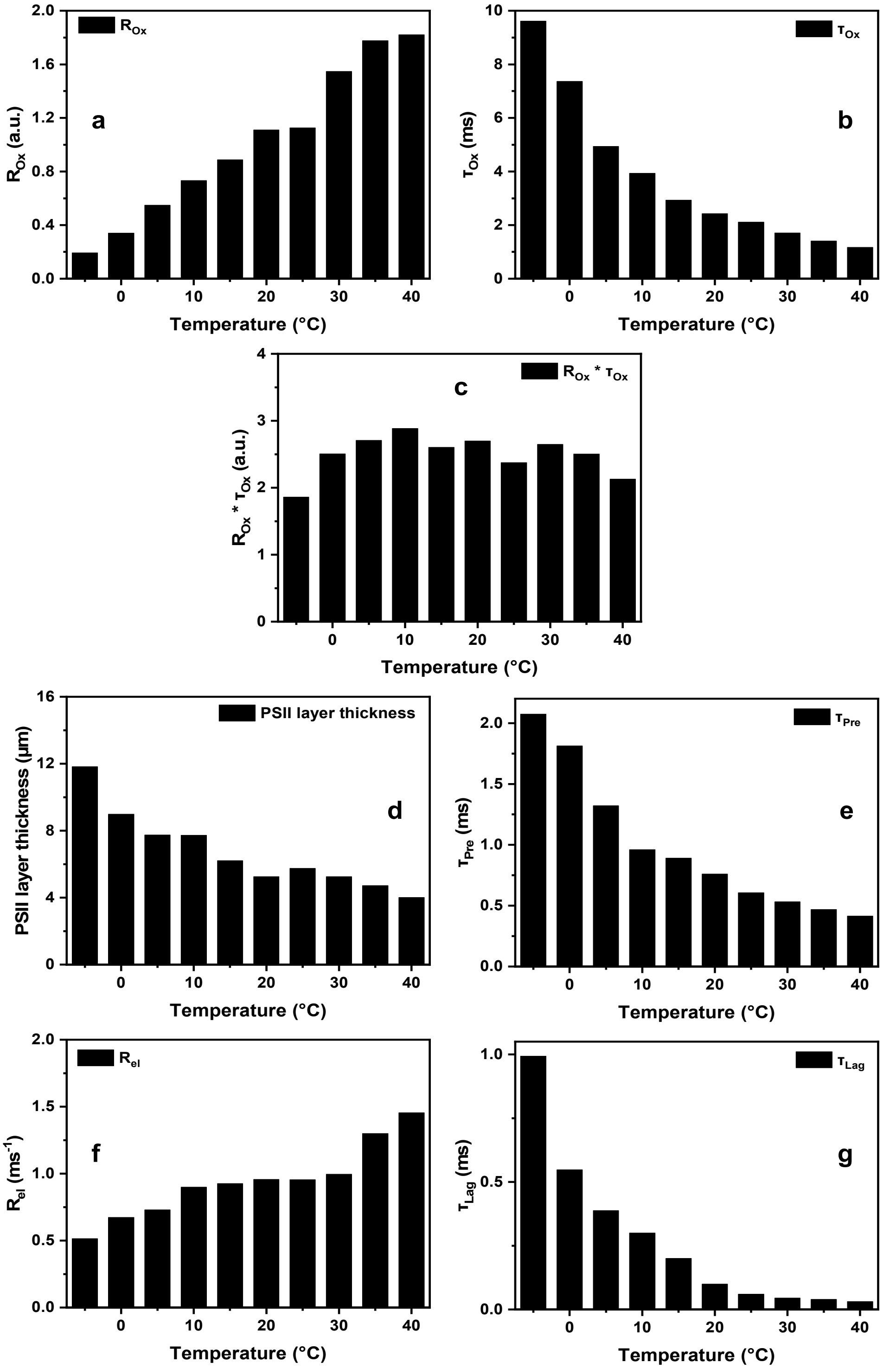

Supplementary Fig. 6. The complete sets of fit parameters in simulation of the O_2-evolution transients of Extended Data Fig. 5. **a**. Parameter that scales with the rate of O_2-formation (R_{ox}). **b.** Time constant (inverse rate constant) of oxygen evolution by PSII (τ_{O2}). **c**. Product of R_{ox} and τ_{O2}, a quantity that is proportional to the flash yield of O_2-formation (proportional to the total amount of O_2 molecules that are formed). The O_2 flash yield has been found to be slightly temperature dependent only, with maximal flash yield (minimal miss parameter) around 10°C,[86] in the line with the behavior visible in panel c. This agreement between fit parameters and expected behavior strengthens the confidence in

the simulation results. **d.** Apparent thickness of the layer of PSII membrane particles deposited by centrifugation on the bare platinum electrode, assuming tabulated values for the O_2-diffusion constant of pure water. The seeming temperature dependence of the layer thickness reflects the temperature dependence of the O_2 diffusion constant of the layered PSII membrane particles. **e** and **g** show the parameters τ_{pre} and τ_{lag}, which describe the delayed onset of the O_2-evolution transients. To both, τ_{pre} and τ_{lag}, the delayed onset of O_2-formation by PSII as well as the electrode response contribute. **f**. The R_{el} parameter corresponds to the rate constant of O_2 reduction at the bare platinum electrode, which increases with increasing temperature.

SI.8 Time constants in simulation of S-state transitions

Supplementary Tab. 2. Fit parameters for the transients shown in Fig. 1 b and Extended Data Fig. 2, for all four S-state transitions. Time constants marked with * were set constant while all others were allowed to vary. Due to the fit ansatz described in the Materials and Methods section, the offset represents the starting value of the transients immediately after the flash (t = 0 s). Amplitudes and offset values are provided in µOD.

S_1 -> S_2	Offset	33 µs		91 µs	3.1 ms	25 ms
1384 cm^{-1}	110	-31		-72	10	11
1402 cm^{-1}	76	1.0		-180	-1.7	19
1449 cm^{-1}	8.4	-15		58	6.5	-28
1568 cm^{-1}	33	-57		-110	-15	58
1706 cm^{-1}	-17	-73		55	-17	36
S_2 -> S_3		43 µs		290 µs	1.8 ms	23 ms*
1384 cm^{-1}	-14	52		-59	14	2.3
1402 cm^{-1}	77	-22		-56	44	22
1449 cm^{-1}	93	-98		77	14	-5
1568 cm^{-1}	-110	172		-100	34	22
1706 cm^{-1}	55	-140		11	66	8.5
S_3 -> S_0		26 µs	65 µs	340 µs	2.5 ms	22 ms
1384 cm^{-1}	105	-3.3	-98	0.3	73	-4.9
1402 cm^{-1}	93	-120	3.4	59	71	6.4
1449 cm^{-1}	61	-5.7	-12	-24	-27	-12
1568 cm^{-1}	-130	57	-26	80	-58	64
1706 cm^{-1}	51	-41	-27	-100	127	-3.5
S_0 -> S_1		28 µs		150 µs	1.5 ms	23 ms*
1384 cm^{-1}	21	-60		-31	26	6.6
1402 cm^{-1}	34	-6.5		-13	16	7.0
1449 cm^{-1}	90	-70		-29	19	7.5
1568 cm^{-1}	-48	58		20	-48	8.4
1706 cm^{-1}	79	-52		-100	51	24

Supplementary Tab. 3. Time constant values for S-state cycle reactions from previous investigations. The first τ-column provides the time constant values determined in the respective study at the measurement temperature indicated in the second column. The corresponding value at 10 °C were calculated from the first-column values using the respective activation energy as reported in ref. 11; they are provided in the third column (τ_{10}). Exclusively time constant values are listed which we previously determined using the same protocols for preparation of high-activity PSII membrane particles as used in the present investigation. Comparison of curve-fit results from the various studies suggests a sizeable uncertainty range. Within the limits of the uncertainty present also in the previously reported values, the time constant values of the present investigation agree satisfactorily well with previously reported results.

	**τ**	**Temperature \°C**	**τ_{10}** (at 10°C)	**Method**	**Reference**
S_1 -> S_2	120 µs	10	120 µs	photothermal	ref. 11
	100 µs	23	133 µs	UV-visible	ref. 12
S_2 -> S_3	60 µs	10	60 µs	photothermal	ref. 11
	25 µs	RT (25)	66 µs	X-ray absorption	ref. 15
	320 µs		673 µs	X-ray absorption	
	18 µs	10	18 µs	delayed fluorescence	ref. 87
	100 µs		100 µs	delayed fluorescence	
	330 µs		30 µs	delayed fluorescence	
	280 µs	23	535 µs	UV-visible	ref. 12
S_3 -> S_0	300 µs	RT (25)	435 µs	X-ray absorption	ref. 10
	1.6 ms		2.6 ms	X-ray absorption	
	14 µs	20	18 µs	delayed fluorescence	ref. 88
	65 µs		84 µs	delayed fluorescence	
	200 µs		257 µs	delayed fluorescence	
	170 µs	23	235 µs	UV-visible	ref. 12
	1.7 ms		2.6 ms	UV-visible	
	25 µs	20	32 µs	photothermal	ref. 89
	250 µs		322 µs	photothermal	
	150 µs	RT (25)	217 µs	X-ray absorption	ref. 15
	1.6 ms		2.6 ms	X-ray absorption	
	20 µs	10	20 µs	delayed fluorescence	ref. 87
	100 µ		100 µs	delayed fluorescence	
	400 µs		400 µs	delayed fluorescence	
	2.4 ms		2.4 ms	delayed fluorescence	
	2.8 ms	10	2.8 ms	photothermal	ref. 11
	2.2 ms	10	2.2 ms	delayed fluorescence	ref. 88
S_0 -> S_1	50 µs	RT (25)	55 µs	X-ray absorption	ref. 15
	160 µs	10	160 µs	photothermal	ref. 11

SI.9 Spectrokinetic signatures of carboxylate sidechain deprotonation and potentially overlapping Tyr_Z contributions

The IR difference spectra assigned to formation of Tyr_Z^{ox} comprise a dominating negative band at 1706 cm^{-1} and two minor negative bands at 1724 cm^{-1} and 1738 cm^{-1}, and positive bands at 1730 and 1748 cm^{-1} (see inset Figure S9), [90] which raises the question whether and to what extent they contribute to the DAS of Figure 2 (inverted amplitudes are expected for Tyr_Z reduction).

Figure S7 shows an analysis of the data in form of population associated spectra, calculated from the 340 µs and 2.5 ms DAS. Aside from dominating contributions we assign to reversible carboxylate deprotonation, we detect a component assignable to Tyr_Z reduction by electron transfer in the S_3->S_4->S_0 transition. At 1707 cm^{-1}, we estimate a Tyr_Z contribution around 40% to the millisecond DAS and around 6% to the microsecond DAS. The deconvolution in a carboxylate deprotonation component and Tyr_Z related component is straightforwardly done (no adjustable parameters) and it gains credibility by the almost perfect match of the vibrational frequencies previously found in the Tyr_Z^{ox}/Tyr_Z difference spectrum. The limits of this analysis show up in the spectra of State A where instead of a clear peak around 1721 cm^{-1} a fuzzy broad band is obtained, likely explainable by especially high sensitivity of the calculated State-A population to imprecision (noise) in the 340 µs DAS.

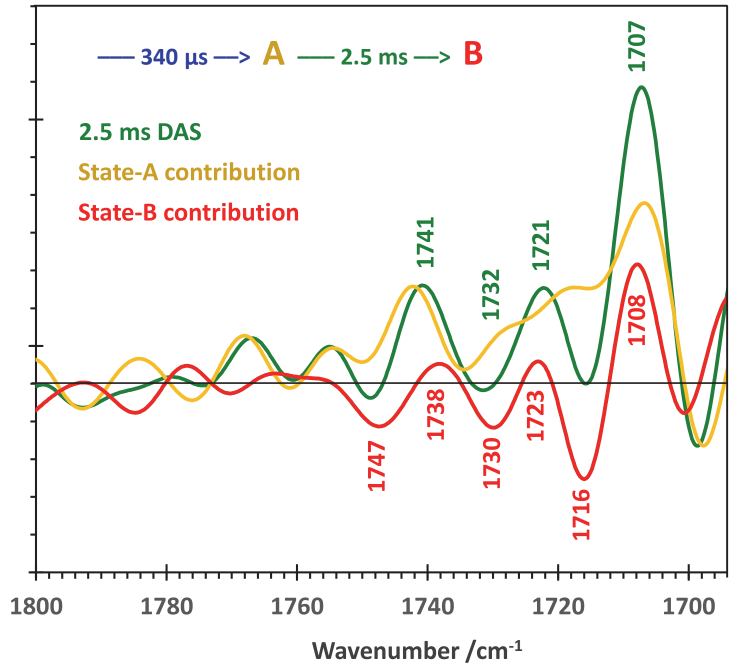

Figure S7. Population-associated spectra discriminating between difference spectra of the transiently formed state (orange) and for the formation of the final state (red). The shown spectra provide the contributions of State-B formation (red) and State-A decay (orange) to the total 2.5 ms DAS (green). State A is transiently formed by proton removal from the PSII donor side, in the presence of Tyr_Z^{ox}. The difference spectrum of State A ($Tyr_Z^{ox}S_3/Tyr_Z^{ox}S_3'$ difference spectrum) is shown in black and assigned by us to deprotonation of a carboxylate sidechain in different H-bonding configurations. The spectrum of State B corresponds to the difference spectrum of the oxygen-evolution transition, during which Tyr_Z is reduced and O_2 is formed. The peak and trough wavenumbers of the State B spectrum match very well the peak and trough wavenumbers expected for Tyr_Z reduction (see inset in Figure S9). Based on this analysis we conclude that the DAS of the spectra shown in Figure 2 reflect predominantly the reversible deprotonation process, but also comprise a significant contribution that corresponds to the

difference spectrum expected for Tyr_Z reduction. From the peak magnitudes at 1707 cm^{-1}, we estimate that this contribution amounts to about 40% of the millisecond DAS and 6% of the microsecond DAS (at 1707 cm^{-1}).

The 340 µs/2.5 ms reversibility in the IR transients is observed at 1707 cm^{-1} (and for the two minor DAS peaks at 1722 cm^{-1} and 1744 cm^{-1}, see Extended Data Figure 4). This reversibility is not observed at other prominent peaks of the Tyr_Z^{ox}->Tyr_Z difference spectrum, as shown in Figure S8, confirming assignment a process unrelated to Tyr_Z oxidation.

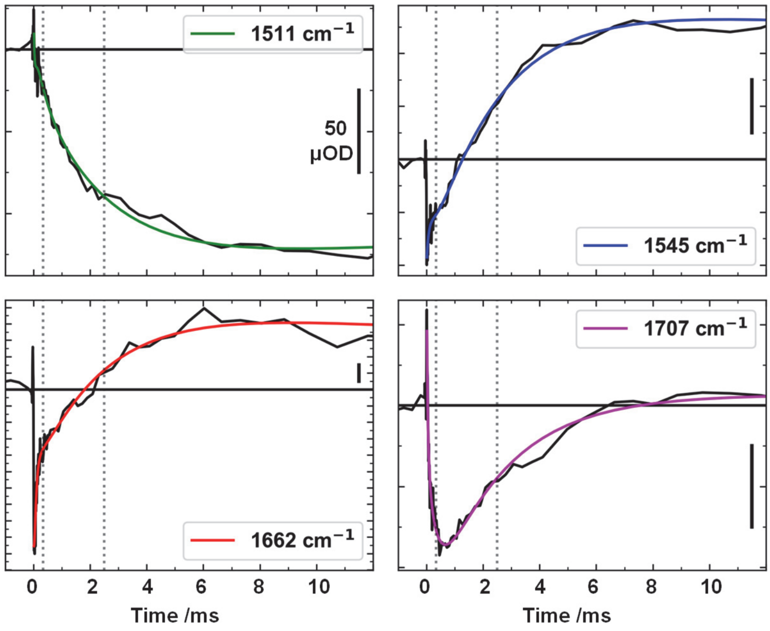

Figure S8. Infrared time courses at prominent wavenumbers of the Tyr_Z^{ox}/Tyr_Z difference spectrum reported by Sato et al. [90] (black lines connecting data points; colored lines simulation corresponding to the decay associated spectra (DAS) of Figure 2. *Only at 1707 cm^{-1} we find a decay in the 350 µs phase that is largely reversed in the 2.5 ms phase.*

[We note that the direction of the putative Tyr_Z^{ox} band is as expected, that is, a decrease at 1511 cm^{-1} and an increase at 1545, 1662 and 1707 cm^{-1}, but we do not see the expected reversal to the zero level. At 1545 cm^{-1} (amide II) and 1662 cm^{-1} (amide I) this may be explainable by other influences on the amide vibrations aside from Tyr_Z oxidation. At 1511 cm^{-1}, however, we currently do not see a similarly straightforward explanation for the unexpected behavior. We speculate that either this Tyr_Z^{ox} band is strongly shifted in PSII with an intact Mn_4Ca complex or that also the 1511/1515 cm^{-1} Tyr_Z^{ox} band is dominated by an electrochromic effect.]

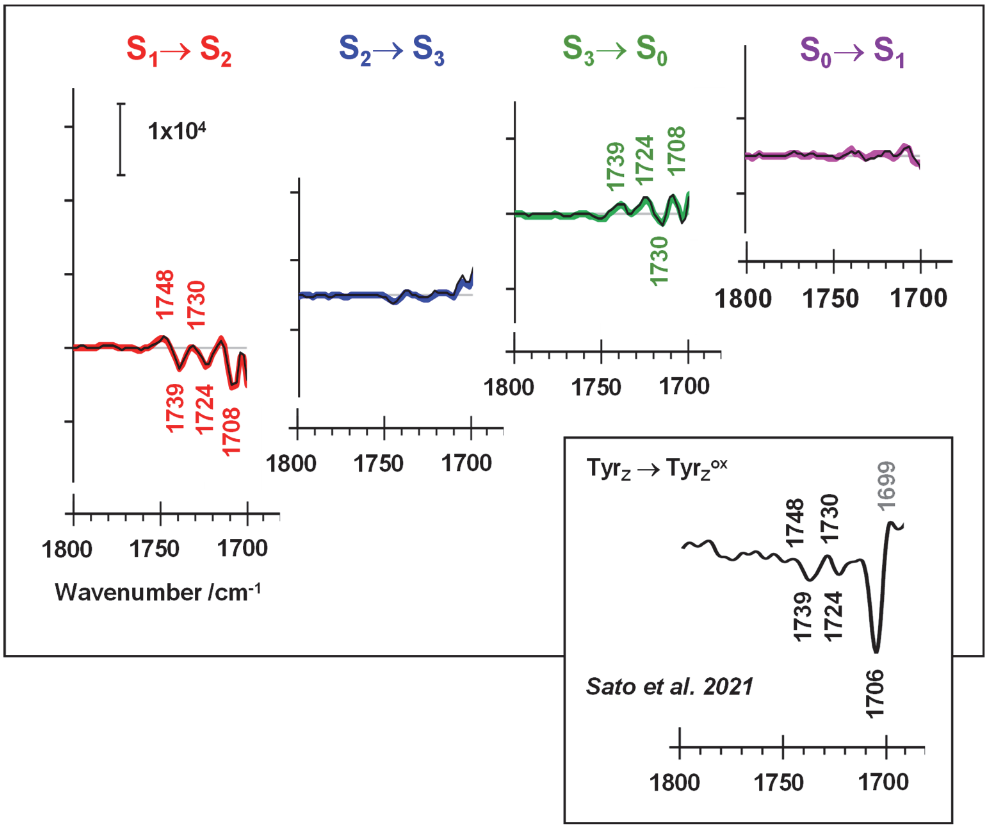

Figure S9. S-state IR difference spectra in the wavenumber range from 1700-1800 cm^{-1}. The FTIR spectra were collected at PSII membrane particles of spinach with a spectral resolution of 4 cm^{-1} (at 10°C, flash-spacing of 3 s, S-state difference spectra obtained by deconvolution as described in SI.5; data collected by Julia Menzel[91] and evaluated by Dr. Petko Chernev). The inset shows Tyr_Z^{ox}/Tyr_Z differences spectra of Sato et al. [90] obtained for Mn-depleted PSII particles of spinach (modified from ref. 90) .

We also observe the Tyr_Z related peaks at 1706, 1724 and 1739 cm^{-1} as negative bands in the S_1->S_2 difference spectrum and as positive bands in the S_3->S_0 spectrum (Figure S9, see also Figure S7), with amplitudes comparable to the Tyr_Z-related spectrum of Figure S7, which on first glance is implausible, because Tyr_Z^{ox} is formed only transiently in the course of an S-state transitions. A revealing explanation is presented in the following.

In seminal studies Noguchi and coworkers convincingly assigned the 1707 cm^{-1} band in the Tyr_Z->Tyr_Z^{ox} difference spectrum to the keto-group of Chl_{D1}, a specific reaction-center chlorophyll of PSII. [90,92,93] The Chl_{D1} is located at a distance of about 10 Å from the proton in the Tyr_Z^{ox}-H-His190 moiety, which gets positively charged upon Tyr_Z oxidation. The resulting electric field causes an electrochromic shift of the Chl_{D1} keto-group band (vibrational Stark effect) associated with a bleaching (negative IR absorption peak) at 1707 cm^{-1}. The other Tyr_Z/Tyr_Z^{ox} bands above 1700 cm^{-1} presumably also relate to electrochromic shifts of vibrational frequencies, but a definitive assignment to specific groups has not been achieved so far.

We now propose that Tyr_Z oxidation and 'positive charging' of the Mn_4Ca-oxo cluster in the S_1->S_2 transition (see refs. 9,50,94) result in similar electrochromic effects, thereby explaining that the S_1->S_2 steady-state difference spectrum above 1700 cm^{-1} resembles closely the difference spectrum reported for Tyr_Z oxidation. [90] In the S_3->S_0 transition discharging of the Mn_4Ca-oxo cluster occurs in the 2.5 ms

phase,[9,50,94] by proton release relating to water binding and metal-oxo cluster reorganization after O_2 formation (see also Extended Data Figure 8), which explains that the S_3->S_0 steady-state difference spectrum above 1700 cm^{-1} resembles closely the difference spectrum of Tyr$_Z$ reduction. These absorption changes are formed concomitantly with the 2.5 ms process and prevail also once the stable S_0-state has formed. It is one consequence of the outlined rationale that the magnitude of the 1707 cm^{-1} peak in the steady-state difference spectra of the S_1->S_2 or the S_3->S_0 transition provide an independent estimate of the strength of the contribution of these bands to the 2.5 ms DAS (resulting in an estimate of 25-45% for the 1707 cm^{-1} band).

The 100 µs spectrum shown in Extended Data Figure 3 represents a good starting point for discussion of the possible contributions of the Tyr$_Z^{ox}$ band above 1700 cm^{-1} at early times in the S_3->S_0 transition. The 100 µs spectrum is better suited than the 10 µs spectrum because in the latter likely is affected by contributions from the oxidized primary chlorophyl donor, P680$^+$, resulting in the positive peak at 1720 cm^{-1} with a shoulder at ca. 1710 cm^{-1} and a negative peak at 1700 cm^{-1} (compare refs. 92,93). These P680$^+$ contributions, which would complicate the discussion unnecessarily, have disappeared in the 100 µs spectrum. At 100 µs in the S_3->S_0 transition, the oxidized Tyr$_Z$ thus should be present in the vast majority of PSII. However, the expected negative band at 1706 cm^{-1} is *not* detectable, which is explained as follows: When Tyr$_Z^{ox}$ is oxidized, the 1706 cm^{-1} bleaching is already present due to the positive charge accumulated in the S_1->S_2 transition.[9,50,94] Therefore, Tyr$_Z^{ox}$ formation may cause an additional shift of the already shifted 1706 cm^{-1} band, but it cannot enhance the electrochromic bleaching at 1706 cm^{-1}. This explains straightforwardly the lack of this electrochromic Tyr$_Z^{ox}$ feature around 1706 cm^{-1} in the 100 µs spectrum of the Extended Data Figure 3. Also, the subsequent deprotonation step associated with the 350 µs DAS should neither enhance the amplitude nor remove the electrochromic 1706 cm^{-1} bleaching.

In conclusion, the above rationale explains plausibly the experimentally detected contribution of Tyr$_Z^{ox}$/Tyr$_Z$ bands above 1700 cm^{-1} to the 2.5 ms DAS, their presence in the steady-state difference spectra as well as their absence at early times in the time-resolved spectra of the S_3->S_0 transition. It allows for an assessment of the magnitude of their contribution to the 2.5 ms DAS, which also is supported by data shown further below in Figure S10 and Figure S11. The contribution to the 2.5 ms DAS is significant, but not dominating; the contribution of the 1706 cm^{-1} band to the DAS of the 350-µs phase is expected to be negligibly small (well below 10%).

SI.10 Comparison of DAS for data sets collected in deuterated water

Collection of D_2O step-scan data set – Materials & Methods

A step-scan data set was collected for PSII membrane particle immersed in a buffer with H_2O replaced by D_2O. Otherwise the infrared samples were prepared as described further above. The preparation of the roughly 40 CaF_2 samples for a single instrument loading and their adjustment to the desired optical density (1 OD in the amide-I region) took place in air. Therefore, it was not possible to avoid contaminations by H_2O vapor. Based on the relative amplitudes of the D_2O band at 1210 cm^{-1} and the HDO band at 1440 cm^{-1} (amplitude ration of 1.1), we estimate that the fraction of protons in the deuterated PSII samples was 35% (41% D_2O, 46% HDO, 13% D_2O).

The thicknesses of the layer between CaF_2 plates and thereby the PSII amount of H_2O and D_2O samples were adjusted to unity absorption in the amide-I region (at 1650 cm^{-1}). Due to major contributions H_2O vibrations around 1650 cm^{-1} in the non-deuterated IR samples, the unity-adjustment of the OD at 1650 cm^{-1} resulted in a significantly increased PSII amount in the D_2O experiment and thus increased amplitudes of difference spectra.

The D_2O spectra were rescaled to match the amplitudes of the H_2O spectra using two wavelength-independent numbers, s_L and s_A, according to the following equation:

$$\Delta A(\nu) = -\frac{1}{\ln(10)} \frac{s_L \, \Delta I(\nu)}{s_A + I_{total}(\nu)},$$

where $\Delta A(\nu)$ is the absorption difference spectrum, $\Delta I(\nu)$ the flash-difference spectrum of the transmitted IR-light intensity, and $I_{total}(\nu)$ the total spectrum of the transmitted IR-light intensity before (or after) the laser flash. The rescaling by s_L accounts for the increased amount of PSII in the D_2O samples whereas the rescaling by s_A (negative for optimal D_2O rescaling) addresses different extents of thickness inhomogeneities ($s_L = 1$ and $s_A = 0$ for calculation of H_2O spectra from $\Delta I(\nu)$ and $I_{total}(\nu)$; approximation valid for $\Delta I(\nu) \ll I_{total}(\nu)$).

We focus on comparison of the H_2O and D_2O data of the deprotonation phase ($Tyr_Z^{ox}S_3$->$Tyr_Z^{ox}S_3$', 340 µs/700 µs in H_2O and D_2O, respectively) and the O_2-evolution phase ($Tyr_Z^{ox}S_3$'->Tyr_ZS_0; 2.5 ms/3.4 ms in H_2O and D_2O, respectively). To eliminate heat artefacts as well as acceptor side contributions, we directly subtracted the S_1->S_2 data from the S_3->S_0 data (at all wavenumbers and times, without additional scaling). This simple approach can provide a direct comparison of reaction step at the PSII donor side in the S_3->S_0 transition in the time domain above 200 µs because the donor side events in the S_1->S_2 transition are completed with time constants around 100 µs. This analysis approach was chosen mostly because it avoids the heat artefact correction, which is especially intricate in the deuterated PSII samples with still relevant H_2O contributions. Figure S10 illustrates the reassuringly good agreement of H_2O and D_2O data in parts of the spectral range and demonstrates the successful suppression of acceptor side contributions.

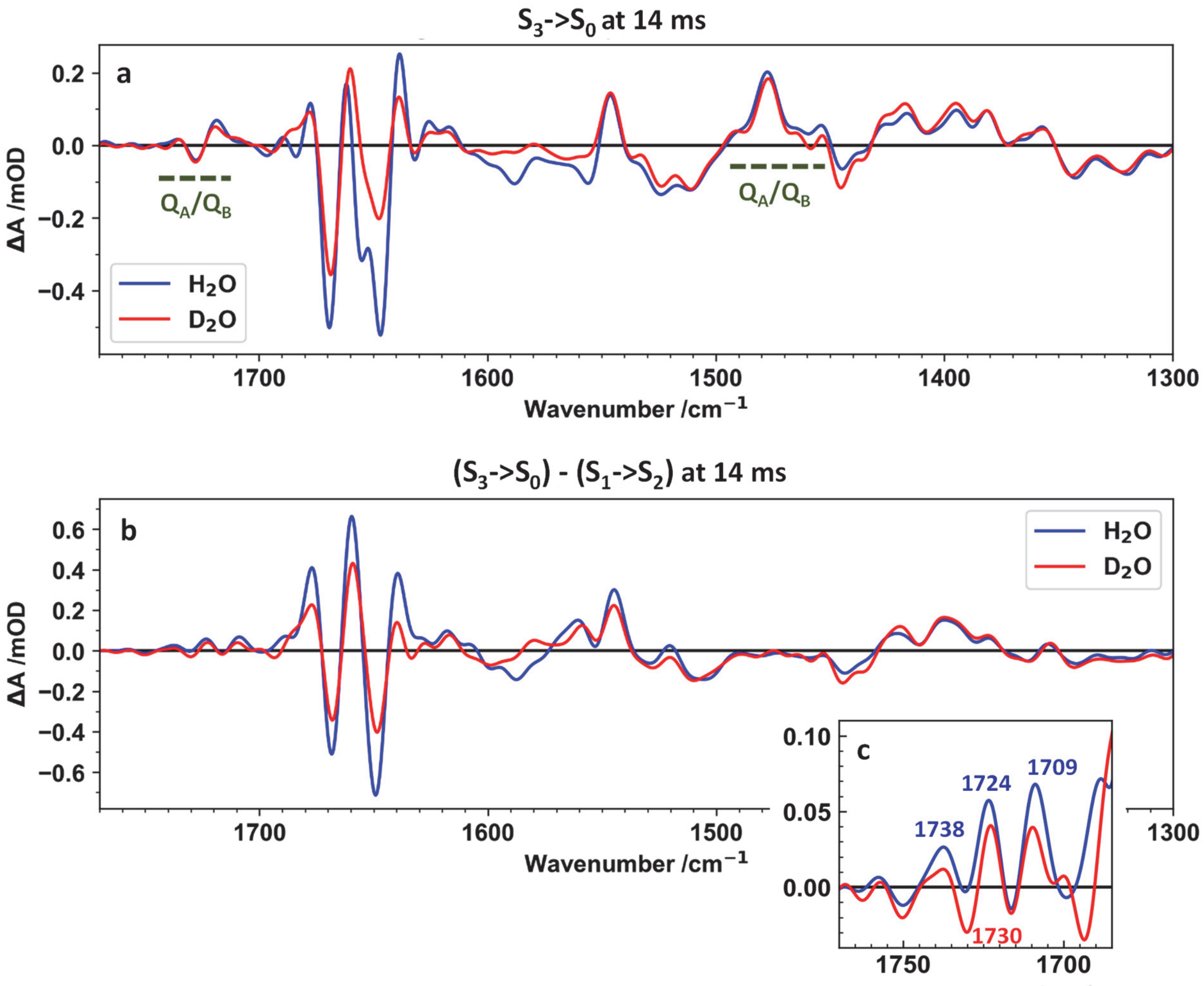

Figure S10. Difference spectra for PSII in H_2O and D_2O at 14 ms. **a)** Difference spectra at 14 ms (10-18 ms average) in the S_3->S_0 transition. **b)** The spectrum of the S_1->S_2 transition was directly subtracted from the S_3->S_0 spectrum (without employing a weighting factor), thereby eliminating bands assignable to the quinone redox chemistry of Q_A and Q_B. **c)** Zoom of the spectral region above 1675 cm^{-1}. The spectrum shown in panel-c resembles closely the corresponding spectrum at later times (not shown). We note that because of the (S_3->S_0)-(S_1->S_2) subtraction, the inverted features of the respective S-state difference spectra in Figure S9 add up, resulting in roughly twice the amplitude of the 1708-band. Based on Figure S9 and panel-c, we estimate the magnitude of the 1708-band as formed in the S_3->S_0 transition to be around 40 µOD (80 µOD/2 for the H_2O data set, which corresponds to about 30% of the DAS amplitude).

Comparison of H_2O and D_2O data sets

Decay associated spectra were determined (Figure S11) and analyzed with focus on the bands in the main article assigned to reversible carboxylate sidechain deprotonation (Figure S10 c).

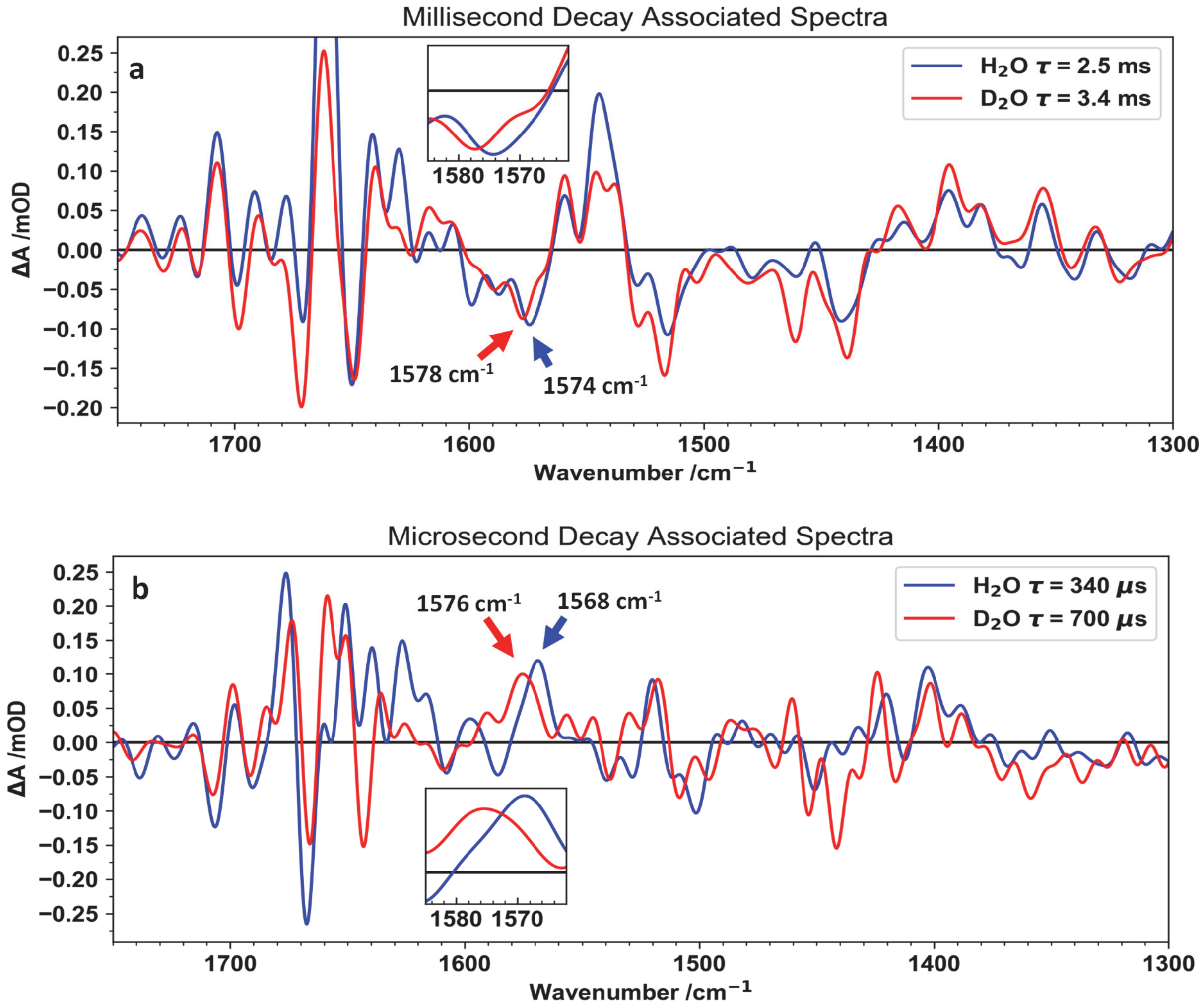

Figure S11. Decay associated spectra (DAS) of the (S_3->S_0)-(S_1->S_2) data set for PSII in H_2O (blue lines) and D_2O (red lines). To address the S_3->S_0 transition, all IR transients were simulated in the time domain from 100 µs to 20 ms only, because in this time-range no major donor-side specific kinetics are expected for the S_1->S_2 transition. The H_2O-DAS of the millisecond phases (in **a**) resembles closely the corresponding DAS of Figure S13 reassuringly well. The microsecond DAS' should be treated with caution. Inter alia because of the limited time range of the fit, the microsecond DAS seems to be reasonably reliable only in regions where the time courses exhibit a visible reversibility (see Figure S12).

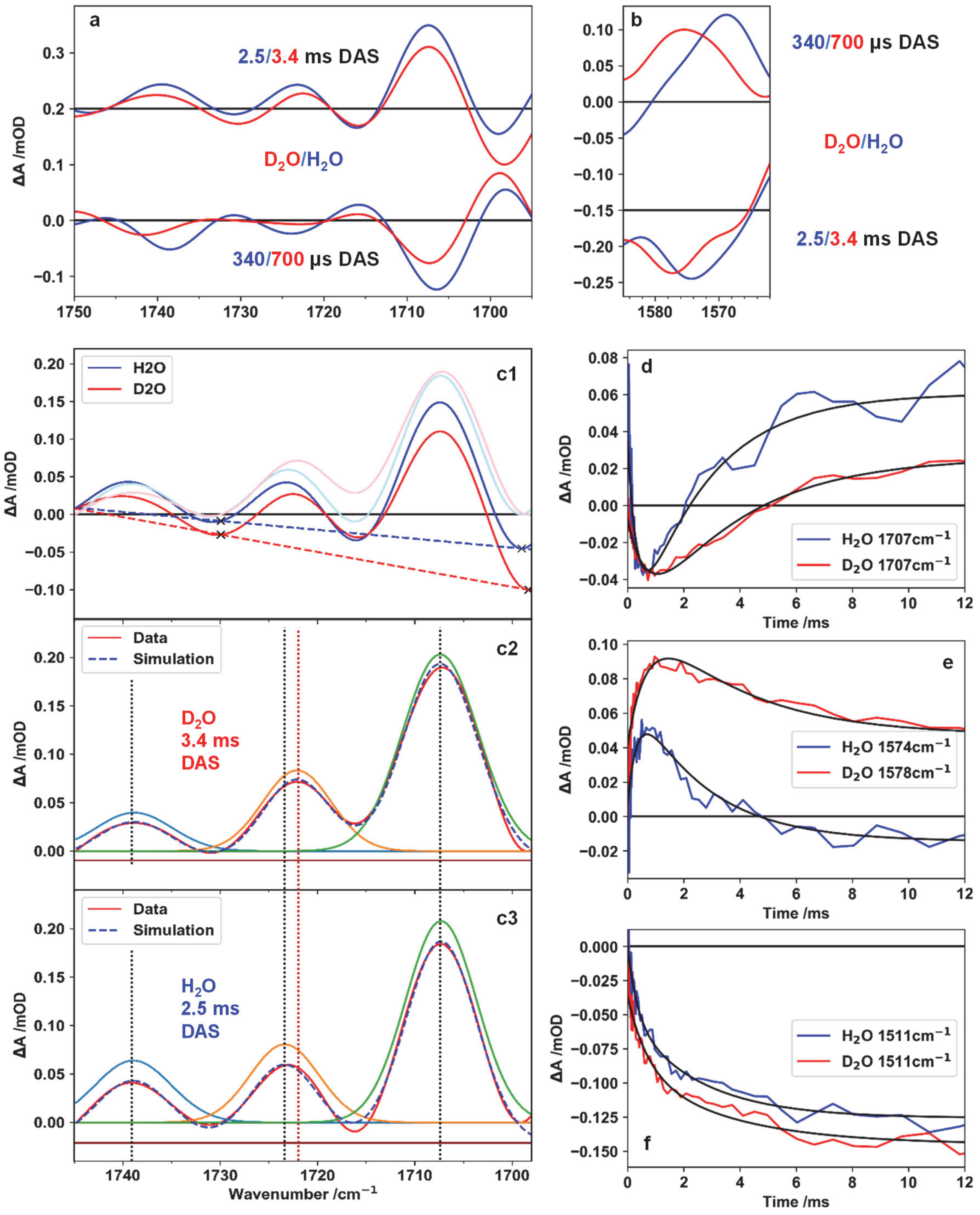

Figure S12. Comparison of decay associated spectra (DAS) and time course of the S_3->S_0 transition in H_2O and D_2O. (**a**) Enlarged presentation of the DAS' shown Figure S11 for features herein assigned to protonated carboxylate sidechains. No clear spectral shifts are resolved, see panel-c for further analysis. (**b**) Enlarged presentation of the DAS' for features herein assigned to asymmetric O-C-O vibrations of carboxylate sidechains. A clear upshift in D_2O by 4-8 cm^{-1} is detected. (**c**) Comparison of peak positions after subtraction of a linear background and simulation with Gaussian functions for the DAS' of the millisecond phases. Wavenumbers of peak position in H_2O/D_2O: 1707 cm^{-1}/1707 cm^{-1}, 1723 cm^{-1}/1722 cm^{-1}, and 1739 cm^{-1}/1739 cm^{-1}. A downshift by 1-2 cm^{-1} in D_2O is revealed by visual inspection of panel-a and confirmed by the simulations; no downshift is detected for the peaks at 1707 cm^{-1} and 1739 cm^{-1}. Alternative analyses approaches involving different background subtraction

modes or inclusion of negative peaks can result in different shifts of peak positions, including a comparably minor downshift of the 1708 cm^{-1} peak of maximally 1 cm^{-1}. Determination of the peak positions were especially uncertain for the 1739 cm^{-1} peak so that here we refrain from any conclusion regarding peak shifts upon D_2O exchange. (**d, e, f**) IR transients at selected wavenumbers. The IR transients for wavenumbers around 1707 cm^{-1} and 1575 cm^{-1} exhibit the reversible behavior predicted for transient (reversible) deprotonation of a carboxylate sidechain. The transient at 1511 cm^{-1} is shown as an example for a non-reversible transient, as it is detected at most wavenumbers.

For the band around 1575 cm^{-1}, we observe a clear upshift in D_2O by about 4 cm^{-1} (from in the millisecond DAS, which seems to correlate with a upshift in the microsecond DAS (Figure 5_e, panel-b). This observation confirms our assignment because a similar upshift of the asymmetric carboxylate frequency was indeed observed with H_2O and D_2O exchange. [95,96]

For the three bands at wavenumbers greater than 1700 cm^{-1}, nearby strong bands below 1700 cm^{-1} (and weak features above 1750 cm^{-1}) in conjunction with baseline uncertainties complicate the analysis. We identify a downshift of the 1723 cm^{-1} band by 1-2 cm^{-1}, but likely no downshift of the 1708 cm^{-1} band ($\Delta\nu$ < 1 cm^{-1}). For protonated carboxylate sidechain in protein, downshifts of 3-7 cm^{-1} have been experimentally detected; [97,98] even larger downshift figures were calculated. [99] In our experiment, (i) incomplete H_2O/D_2O exchange (D/H ratio of ca. 65%/35%, ca. 13% H_2O and 46% HDO) and (ii) a contribution of 25-40% of Tyr_Z^{ox}-related electrochromic kinetics to the millisecond DAS can explain the reduced extent of the downshift.

We propose that the absence of any significant H_2O/D_2O effect on the 1708 cm^{-1} band is explainable by the special character of the proton as a 'shared proton' within a strong H-bond (between Glu65 and Glu312). The frequency is of 1706 cm^{-1} is in line with the assignment to a shared proton. [100] Whether failure of exchange in the only partially deuterated solvent or the special situation as a shared proton explains the H_2O/D_2O exchange insensitivity currently remains open.

SI.11 Deprotonation in $Tyr_Z^{ox}S_3$->$Tyr_Z^{ox}S_3$' transition assigned to Glu65—Glu312 dyad

Our time-resolved IR data on the S_3->S_0 transition in the spectral regime above 1700 cm^{-1} suggests carboxylate-sidechain deprotonation and re-protonation with time constants of 340 µs and 2.5 ms, respectively (700 µs and 3.4 ms in D_2O). Vibrations the protonated forms of glutamate and aspartate sidechain are located in this spectral region and the corresponding vibrational bands disappear upon deprotonation. Their disappearance is expected to be associated with the appearance of 'positive' bands in two spectral regions, (i) 1540-1630 cm^{-1} assignable to the asymmetric carboxylate vibrations and (ii) 1300-1460 cm^{-1} assignable to the symmetric carboxylate vibrations. The identification of the corresponding vibrational frequencies in the here reported DAS is seriously hampered by a multitude of overlapping spectral changes (Figure S11). Especially the six carboxylate residues bound to the metal ions of the Mn_4Ca-oxo cluster give rise to pronounced spectral changes in the S_3->S_0 transition, but also further carboxylate sidechains in the vicinity of the metal-oxo cluster likely contribute (Asp61 and others) as well as the peptide groups of the protein backbone (amide-I and amide-II vibrations). However, only in the absence of major overlap with spectral changes of other groups, the two criteria for identifying vibrational frequencies of transient carboxylate deprotonation are clear-cut:

(i) opposite sign but approximately the same absolute amplitude in the 340 µs DAS and in the 2.5 ms DAS at the respective vibrational frequency;
(ii) only a minor absorption change in the stationary difference spectrum of the S_3->S_0 transition.

We are confident that these two criteria are sufficiently well fulfilled for the DAS bands above 1700 cm^{-1} and the vibrations close to 1570 cm^{-1} (and likely also at 1590 cm^{-1}). The vibrational frequency of 1570 cm^{-1} and signs of the corresponding DAS amplitudes indicate assignment to transient formation of a deprotonated carboxylate sidechain, supported by the frequency shift upon D_2O exchange. (We note at 1590 cm^{-1}, a similar D_2O frequency shift is detected in the millisecond DAS but not in the microsecond DAS, likely relating to a comparable high noise level in the microsecond DAS.) The vibration at 1570 cm^{-1} is assignable to asymmetric carboxylate vibrations and similar spectrokinetic evidence for transient carboxylate deprotonation could come from the symmetric carboxylate vibration. However, we cannot identify vibrational frequencies where the above criteria for clearcut identification of transient carboxylate deprotonation are fulfilled. In other words, we are convinced that also symmetric carboxylate vibrations reflect transient deprotonation but based on the available spectrokinetic data, the corresponding frequencies cannot be identified with sufficient confidence because of too many overlapping non-transient spectral changes.

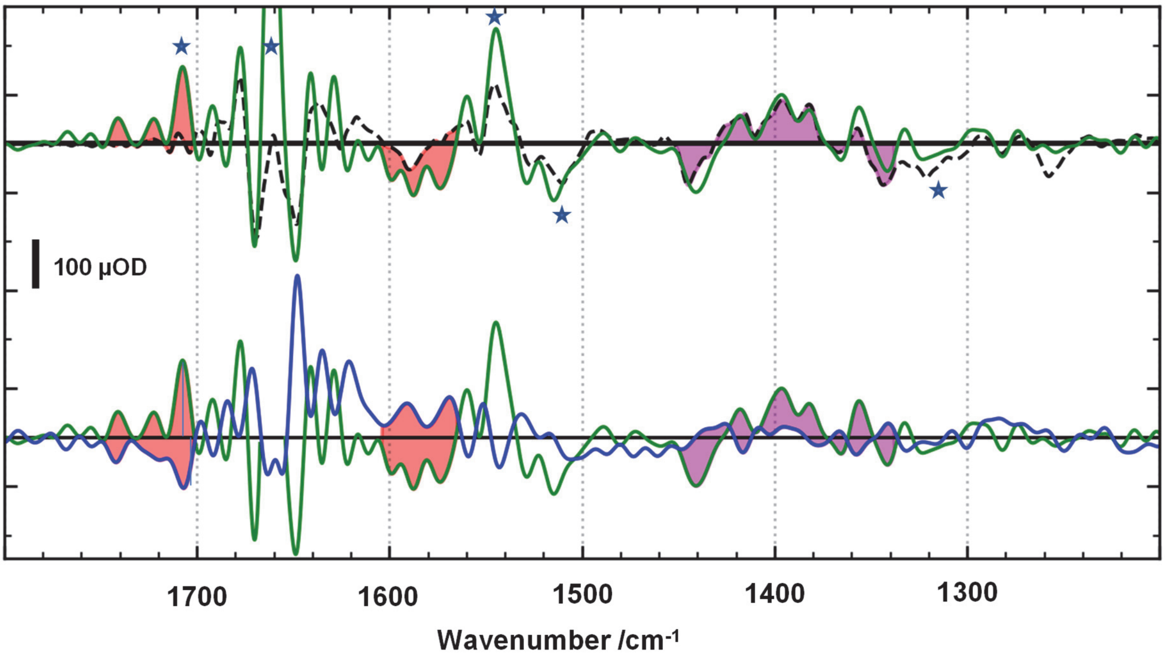

Figure S13. Decay-associated spectra (DAS) of Figure 2 shown for an extended spectral range. Green line, 2.5 ms DAS; blue line, 340 µs DAS; broken line, steady-state difference spectrum of the S_3->S_0 transition as obtained from the multi-exponential DAS simulations (extrapolated value for infinite time); shaded areas as in Figure 2. The blue stars mark the position of possible contributions of Tyr_Z-related vibrations to the 2.5 ms DAS (based on ref. 90).

In past years we have provided evidence that the 340 µs phase is assignable to a deprotonation event that is a prerequisite for the subsequent O_2-formation step. [11,50,89] The here presented time-resolved IR data now facilitates assignment to carboxylate sidechain deprotonation. The assignment to a

specific carboxylate can be made only on basis of high-resolution structural data in conjunction with computational chemistry, as described in the following. On a first glance transient deprotonation of Asp61 appeared to us as a plausible assignment. Our analysis presented in the section SII, however, clearly shows that this residue is deprotonated in the S_3 state, a conclusion which is in line with previous computational studies, [37-40] and thus its transient deprotonation can be excluded. We also did not identify any other likely protonated Glu/Asp residue in in vicinity of Tyr_Z and the Mn_4Ca-oxo cluster, whereas for the Glu65-Glu312 pair there hardly can be any doubt that this residue pair is protonated. Strong H-bonding by a more or less shared proton is indicated by the short H-bonding distance revealed by protein crystallography. [21,25] Whether the proton is preferentially located on one of the two residues, oscillating between the two residues, or delocalized within a low barrier hydrogen bond is still unclear. We favor predominant protonation of Glu312, for reasons explained in section SII.2, whereas others have assumed preferential protonation of Glu65. [43] We emphasize that the specific location of the proton within the Glu65-Glu312 dyad is of little relevance for our mechanistic conclusions.

Previously we have provided evidence that a deprotonation step precedes the electron transfer (ET) step not only in the S_3->S_0 transition, but also in the S_2->S_3 transition, [11,89] Therefore, assuming that the deprotonation step in these two S-state transition are similar, the impressive time-resolved crystallographic results for the S_2->S_3 transition can support also our analysis of the S_3->S_0 transition. In ref. 21 it is shown that in the S_2->S_3 transition the electron transfer step is preceded by moderate conformational changes around the Glu65-Glu312 dyad. A prominent feature is the rotation of the Glu65 residue associated with an increase in the Glu65-O--O-Glu312 distance from 2.46 to 2.72 Å. We detect a similar Glu65 rotation when comparing the protonated Glu pair to situation with unprotonated Glu65 and Glu312 in MD simulations, see Fig. S16. Inter alia since we have shown that there is a deprotonation that proceeds the ET step also in S_2->S_3, we take the reported Glu 65 rotation as evidence for deprotonation of the Glu65-Glu312 dyad. We note that the reported also for the rotated Glu65, the Glu65-O--O-Glu312 distance of 2.72 Å still suggests an H-bonding situation, conflicting with the notion of deprotonated Glu65 and Glu312. However, multiple occupancies could have affected the distance precision significantly; corrections that are analogous to the S-state deconvolution we applied to the IR data set cannot easily be applied to the time-resolved crystallographic data. In conclusion, there are good reasons to assume that the deprotonation steps preceding the ET in the S_2->S_3 and S_3->S_0 transition are similar regarding the involved residues. Therefore, the evidence for transient deprotonation of the Glu65-Glu312 in the S_2->S_3 transition supports our assignment of the transiently deprotonated residue(s) in the S_3->S_0 transition.

Last not least, for deprotonation of the Glu65-Glu312 dyad, we provide a plausible explanation how this specific deprotonation step is induced by Tyr_Z oxidation and how it promotes the proton-coupled ET in step of oxyl radical formation, strongly supported by recent computational results of Kaila and coworkers. [34]

SII. Supplementary Discussion – Computational Chemistry

SII.1 Exploring protonation states of carboxylate groups in the S_3 steady state – Asp61 (D61)

The protonation state of Asp61 in the S3 steady state has been investigated by means of ab-initio geometry optimization of the crystallographic structure resolved by Kern et al. [25], starting with a protonated Asp61 carboxylate and W1 in its hydroxide form, as shown in Supplementary Fig. 14.

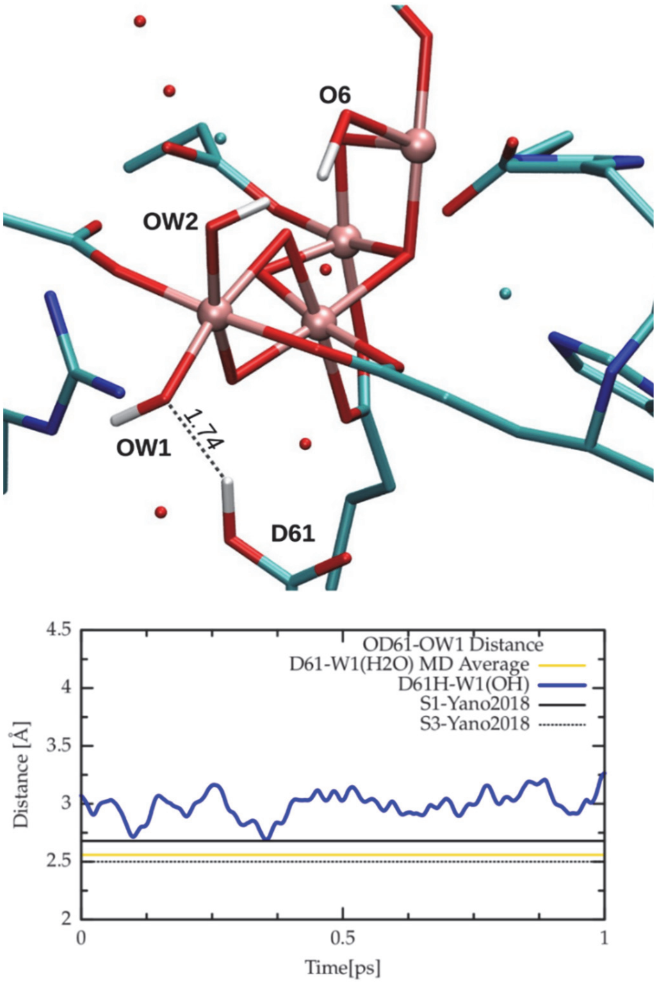

Supplementary Fig. 14. Top panel: Starting positions of the geometry optimization to analyze the reliability of an Asp(D)61 protonation. After few iterations of the geometry optimization the bond between the proton and the carboxylic oxygen of Asp61 is broken, and the water form of W1 molecule restored. Bottom panel: Distance between the hydrogen bonding oxygens of Asp61 and OW1 in a QM/MM MD simulation after 1 ps of distance restraint. The higher value of such distance with respect to x-ray is stable in the picosecond of the free simulation, corresponding to a different hydrogen bonding network. The distance values are compared with the crystallographic distances in S_1 and S_3 from ref. 25 and with the average distance calculated along 10 ps of a QM/MM MD simulation with deprotonate Asp61. [26]

Notably, in the first steps of geometry optimization the proton jumps back to the W1 molecule leaving Asp61 in its deprotonated form, thus indicating the instability of such protonation pattern relatively to

the experimental structure of the S_3 steady state. The assumption made in previous theoretical studies indicating a deprotonated state for Asp61 in different steady states of the Kok cycle, is therefore supported also by the local stability of the hydrogen bond network explored by our ab initio calculations based on the crystallographic positions of the S_3 state. Additionally, to overcome local energy barriers and check other possible energy minima corresponding to a protonated form of Asp61, we also performed 1 ps of QM/MM MD simulation, restraining the proton to be bound to Asp61. After 1 ps of restrained QM/MM MD simulation, the restrain was removed and the system left free to evolve for one additional ps. In this time the proton remained bound to Asp61 leaving the W1 water molecule in its hydroxide form. Nevertheless, the distance between the carboxylate oxygen of Asp61 and the OW1 oxygen, was found to diverge significantly from that found in the x-ray structure. On the other side, a previous QM/MM MD simulation of the S_3 state, carried out for 10 ps, considering Asp61 deprotonated,[26] showed an average value much closer to the crystallographic value, when compared with the values of the simulation employed with protonated Asp61 (see bottom panel Supplementary Fig. 14).

SII.2 Exploring protonation states of carboxylate groups in the S_3 steady state – Glu65 (E65)

Protein crystallography suggest a short distance between carboxylate oxygen atoms of Glu65 and Glu312 in H-bonding distance of well below 3 Å. Consequently, the H-bonding proton is associated preferentially either with Glu65 or with Glu312. In ref. 43, Ishikita and coworkers assume that Glu65 is preferentially protonated, in line with their calculations. We consider this option as unlikely, due to the evident stabilization of the deprotonated form of Glu65 arising from the interaction with Arg334 and Asn335. See Supplementary Fig. 15, where we report the distances between such moieties as found in the S_3 crystal structure (see also Supplementary Tab. 4). In line with this observation, empirical pKa calculations carried out with Propka software[101] on the S_3 structure resolved by Kern et al., also confirm a preferential deprotonated state for Glu65, while suggest a protonated form for Glu312 (i.e. pK_a = 7.2 for Glu312 and 2.4 for Glu65).

To provide further support for Glu312 protonation, we performed QM geometry optimizations on a larger system including atoms of Lys317, Glu312, Glu65, Arg334, Asn335 and their closest water molecules, as found in the X-ray structure. [25] In Supplementary Fig. 16 the structural effects arising from the protonation/deprotonation of Glu312 are reported and compared with the crystallographic positions.

In our model the absence of a proton in the carboxylate group of Glu312, has a clear impact on the position of the Glu65 side chain, making it to diverge from its crystallographic position, due to the electrostatic repulsion between the two negative glutamate residues (left panel Supplementary Fig. 16). Conversely, the protonation of Glu312 strongly preserves the X-ray positions after Full-QM geometry optimization (right panel Supplementary Fig. 16). In Supplementary Fig. 17 the RMSD of the atomic coordinates of the two models with respect to the crystallographic positions are reported. The largest value is observed for the case of the model with deprotonated Glu312.

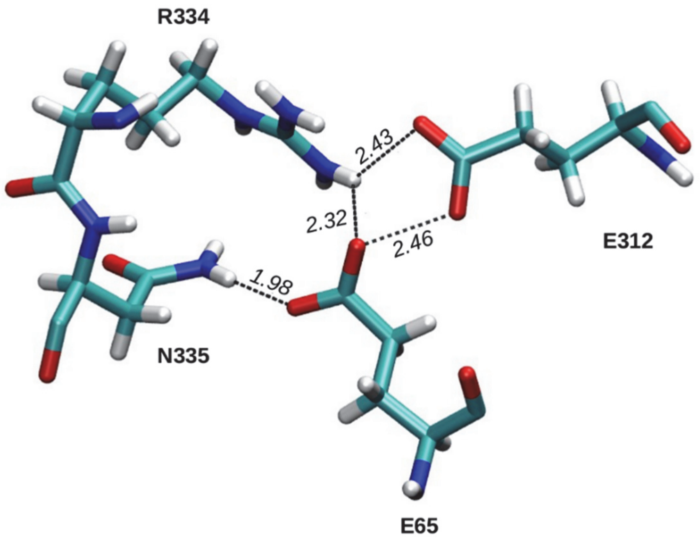

Supplementary Fig. 15. Electrostatic stabilization favoring deprotonated Glu65. The Glu(E)312 – Glu(E)65 – Arg(R)334 – Asn(N)335 sidechain distances as found in the crystallographic data from Kern et al. [25] (pdB 6DHO) are indicated.

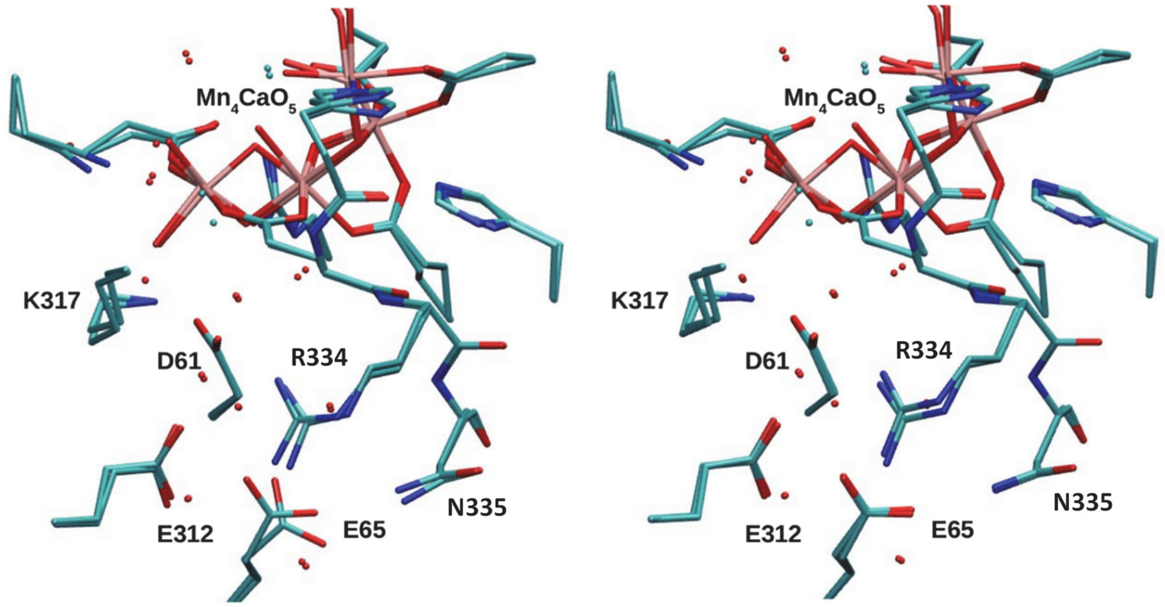

Supplementary Fig. 16. Comparison between X-ray structure[25] and computationally optimized structures. For the sake of clarity, the hydrogen atoms are hidden. The two shown models differ only by the protonation of Glu(E)312. On the left, the computational Glu312-deprotonated model is compared with the X-ray structure (RMSD of 0.315 Å), whereas on the right the Glu312-protonated model is compared with the same X-ray structure (RMSD of 0.271 Å).

Supplementary Tab. 4. Interaction distances of the Glu312 hydrogen bond network.

Distance / State	S_1	S_2	S_3
Glu65-Glu312 (O-O)	2.42	2.55	2.46
Arg334-Glu65 (NH-O)	2.87	2.68	2.32
Arg334-Glu312 (NH-O)	2.35	3.01	2.43
Asn335-Glu65 (NH-O)	2.08	2.04	1.98
Lys317-Asp61 (NH-O)	3.97	3.90	4.16

SII.3 Possible role of Lys317

The possibility of a mechanistically relevant electrostatic interaction between Lys317 and Asp61 is apparent, due to their short distance (see Extended Data Fig. 9a). Electrostatic stabilization of $Lys317^+$ on $Asp61^-$ could prevent an easy and fast protonation of the aspartic residue, therefore preventing its protonation in the S_3 steady state. We assume that deprotonation of Glu312, upon Tyr_Z radicalization, could be coupled to reorientation of the positive Lys317 side chain towards the negative Glu312 side chain. In this way, the deprotonation of Glu312, could promote the subsequent Asp61 protonation, distancing the positive Lys317 side chain from it and therefore increasing its pKa. Alternatively, Tyr_Z radicalization with the formation of the positively charged $Tyr_Z/His190^+$ moiety, could directly induce a reorientation of the Lys317 side chain towards Glu312, thus simultaneously increasing the pK_a of Asp61 and promoting the deprotonation of Glu312.

To support our hypothesis, we show that the side chain of Lys317 indeed can structurally sample conformations strongly interacting either with Asp61 or with Glu312. In this regard we monitored the distances between Lys317 and both Asp61 and Glu312, along 50 ns of two classical MD simulations (Extended Data Fig. 9b,c,d,e). The two simulations differ only for the protonation state of Glu312, deprotonated in Simulation 1 and protonated in Simulation 2, while Asp61 is deprotonated in both. The simulations present an overall stability of the protein structure as shown by the low values of the root mean square deviations (RMSD) of the protein backbone with respect to the starting structure as a function of the simulated time (see Supplementary Fig. 17). Our analysis shows that the side chain of Lys317 preferentially remains close to Glu312 in Simulation 1 (deprotonated Glu312), while preferentially interacting with Asp61 in Simulation 2. Albeit not quantitatively reliable, these results qualitatively show, from a structural point of view, the possibility of Lys317 to potentially interact either with Asp61 or with Glu312, depending on the protonation state of Glu312. Therefore, these findings lead support to a possible role of Lys317 in modulating the protonation/deprotonation of the neighbor residues Asp61 and Glu312 and ultimately the release of the proton from the catalytic center.

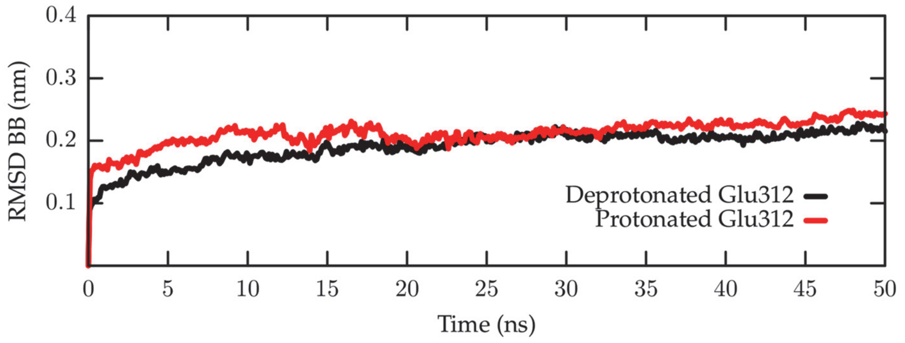

Supplementary Fig. 17. Root Mean Square Deviation of the protein backbone calculated with respect to the starting structure as function of time for the two classically simulated systems: with protonated Glu312 (red line), and with deprotonated Glu312 (black line). In both simulations Asp61 is deprotonated.

SII.4 The minimum energy path of proton-coupled electron transfer (i $\rightarrow$ ii) and peroxo-bond formation (ii $\rightarrow$ iii)

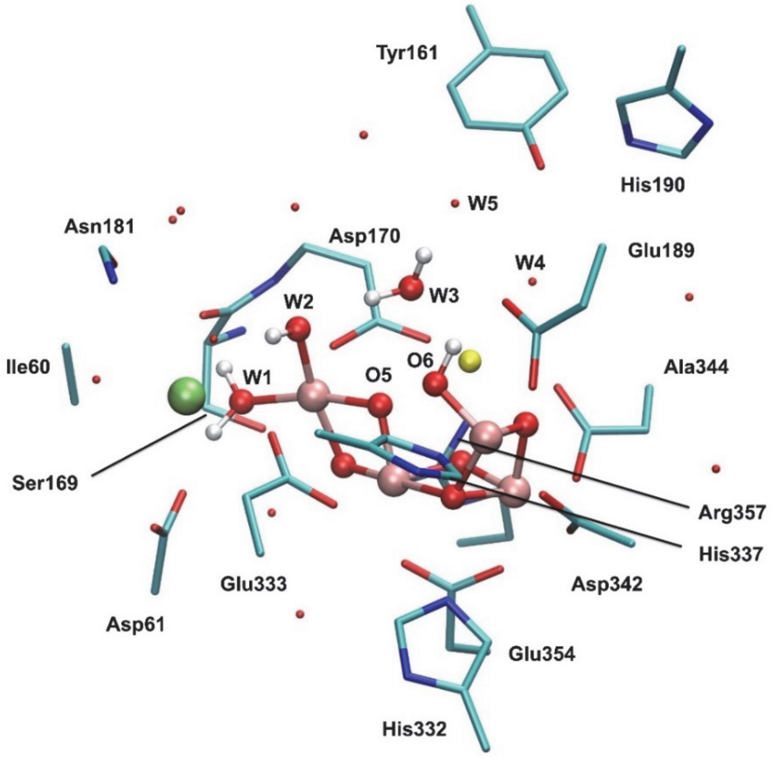

Supplementary Fig. 18. Complete QM region used in the Minimum Energy Path calculation. Representation of all the atoms included in the QM region as discussed in the Method section. All hydrogen atoms not belonging to the $Mn_4Ca(\mu\text{-}O)_5(OH_x)_5$ cluster have been omitted for clarity (color coding: Mn in sepia, Ca in yellow, Cl in green; light atoms in the same standard colors used elsewhere).

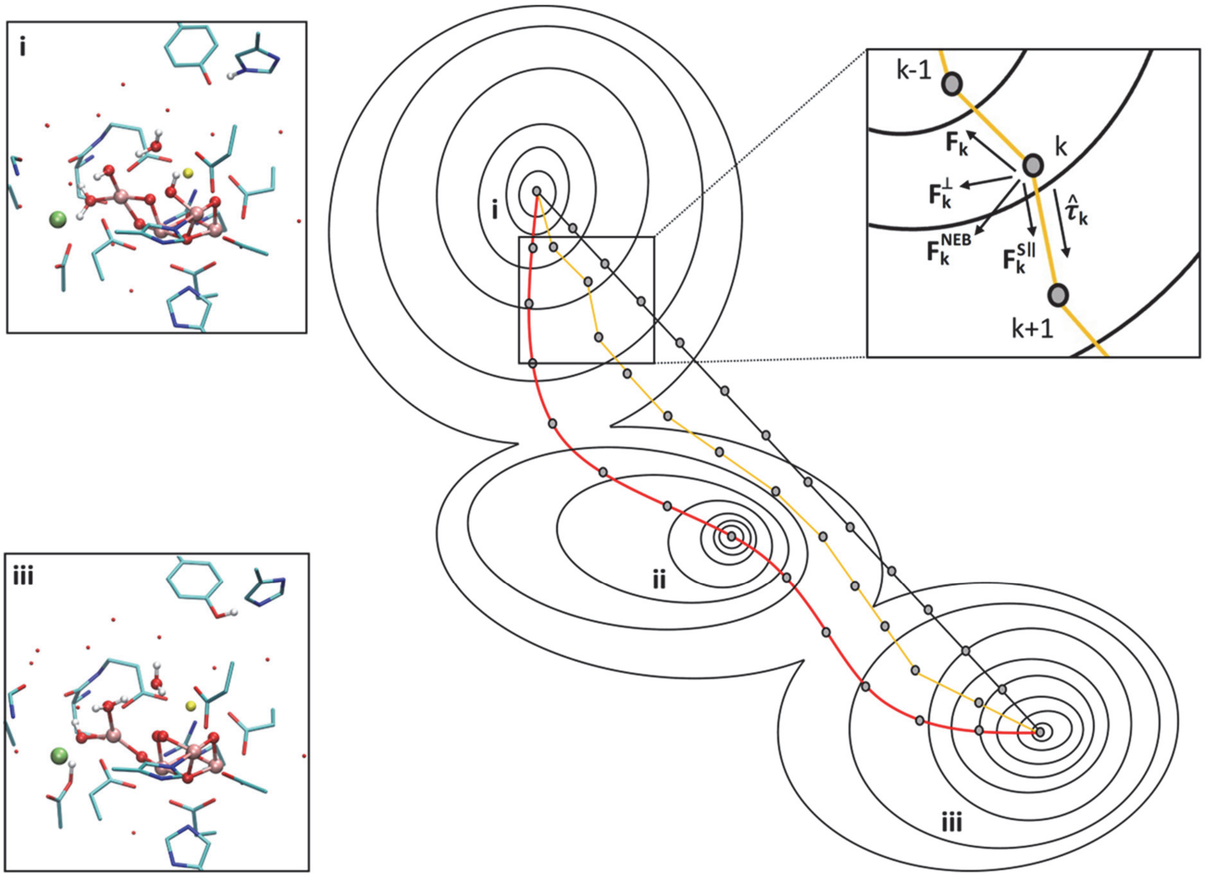

Supplementary Fig. 19. Schematic representation of nudged elastic band (NEB) method employed in the present study in the Minimum Energy Path (MEP) calculations. A set of model structures (here called 'replicas') covering all the reaction coordinate is generated by geometrical linear interpolation between the starting structure (namely, i) and the final structure (namely, iii). Spring forces are applied to connect each model geometry to the two adjacent ones. The energy due to the elastic force and the internal energy of the system are iteratively minimized. The simultaneous minimization of these two energy contributions for all replicas results in structure variation of the individual replicas on the Minimum Energy Path. In the figure, the starting model structures obtained by interpolation are represented by the black line connecting the state i and the state iii. The red curve represents the Minimum Energy Path. The orange curve represents the replicas at a given iteration. In the inset on the top right, the forces taken into account in the NEB calculation are visualized. $F_k^{S||}$ is the spring force along the tangent τ_k, F_k is the force due to the energy potential, and $F_k^{\perp}$ is the respective perpendicular force. The NEB force F_k^{NEB} is calculated starting from its components, $F_k^{S||}$ and $F_k^{\perp}$. The three energy minima are named as i, ii, and iii in accordance with the nomenclature adopted in the present study (see e.g. Extended Data Figure 7).

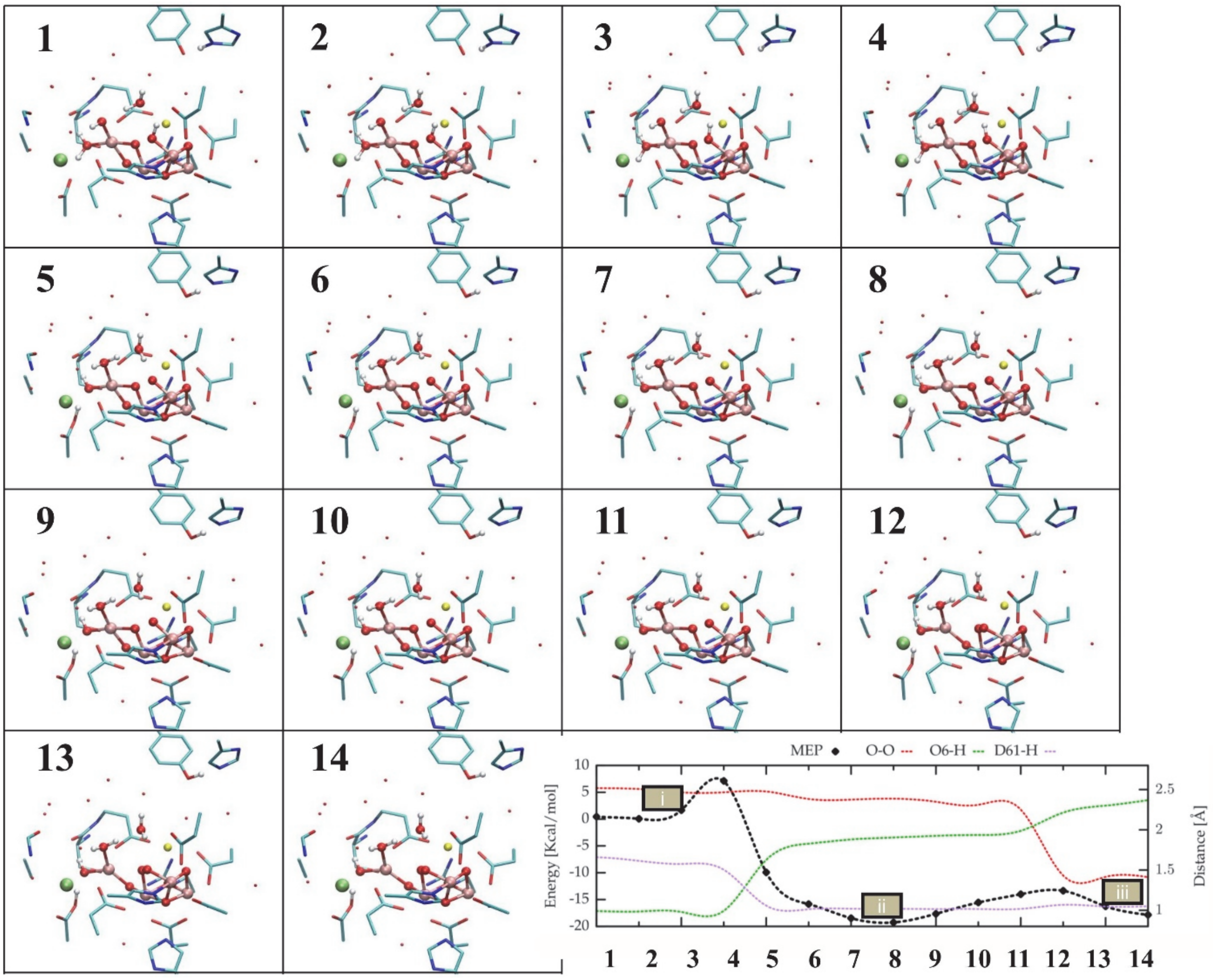

Supplementary Fig. 20. The 14 optimized structures corresponding to the calculated Minimum Energy Path are shown in stick representation together with the respective energy profile, reporting the numbering of each structure. The energy-profile panel also is shown in Figure 3 of the main article. We are aware that differences between the here shown stuctures can hardly be resolved by visual inspection. Therefore, further Supplementary Information on the Minimum Energy Path is provided as separate files:

- As supplementary data, **a separate text file is provided that contains the complete coordinates (incl. H-atoms) of all 14 MEP structures** (PDB data format, the 14 coordinate sets are sequentially listed within the files).
- As supplementary data, **a video is provided (separate mp4 file) that shows the atomic displacement associated with the minimum energy path (MEP) calculation, with focus on the Mn_4Ca-oxo complex and its immediate ligand environment.** The included amino acid residues are shown with labels in Supplementary Fig. 18. The video was obtained for the 14 MEP structures shown in Supplementary Figure 20 by interpolation.

SII.6 Comparison of the present Minimum Energy Path calculations with previous results

A seminal contribution to the computational studies of the molecular details of the mechanisms of PSII water oxidation has been made by P.E. Siegbahn. Already in 2006, well before the acquisition of the first high-resolution X-ray structure of PSII, he proposed a mechanistic representation of the S_3 to S_0 transition with key elements also included in our computational model.[32] Yet in this and further studies of Siegbahn, protons and electrons were jointly or separately removed from the computational scenario without considering explicitly the energy barriers associated with the electron and proton transfer steps. The O-O bond formation mechanism was later refined and a general scheme for all the processes in the Kok-Joliot's cycle was reported by Siegbahn in 2013.[102] More recently, the group of Yamaguchi has provided a major contribution to the picture of the S_3 to S_0 transition.[30, 103] The relation of Siegbahn's and of the Yamaguchi group to our results is discussed in the following, as is a recent study by Kaila and coworkers.[34] Further major computational chemistry contributions were provided by other groups in the field, such as Pantazis,[4,73] Batista,[40] Ishikita,[43] and others, but are not similarly closely related to our investigations.

In contrast to our calculations, in Siegbahn's 2013 study about proton transfer and peroxide bond formation in the S_3 to S_0 transitions,[102] the Tyr_Z/His couple, which provides the oxidation equivalents to the Mn cluster, is not included in the QM region. Although the calculated barrier was numerically similar to the barrier we reported, the proposed proton shift from O6 to W2 was found both without the involvement of Tyr_Z reduction and without the inclusion of W1-Asp61 proton exchange. Therefore, the numerical agreement may be fortuitous (diminished energetics barrier due to neglect of Tyr_Z reduction; increased barrier due to neglect of coupled proton transfer to Asp61). Our computational approach integrates the decisive electron transfer and proton transfer events into a consistent molecular scheme.

Conversely, the Yamaguchi group proposed an extremely high barrier for the proton reorganization around O6 (19 Kcal/mol).[104] This result is disproved by the lower enthalpic contribution, and the non-negligible entropic contribution as they were experimentally determined (Extended Data Figures 5). In their study, a free-energy stabilization from the protein is hypothesized, but without convincing atomistic explanation. For the subsequent step of peroxide bond formation,[103] however, the proposed barrier has very similar value to the one reported here by us, confirming the robustness of the used QM/MEP approach and its consistency with previous calculations.

In both above-cited studies, neglecting the Tyr_Z hydrogen bond participation prevented the rationalization of the important entropic contribution found in our experiments. These cannot be rationalized without considering both the differences of hydrogen bond network (HBN) around radical and neutral Tyr_Z as well as a proper treatment of the proton transport chain from O6 to Asp61.

During the review phase of the present work, an important study was published by the group of Kaila,[34] which convincingly addresses proton and electron transfer steps in the S_2 to S_3 transition. Also, the O-O bond formation in the S_3 to S_0 transition is addressed and the proposed mechanism of proton transfer in the O-O bond formation chemistry shares features with the one reported by us as, but also exhibits major differences: (i) A significant difference regarding the proposed proton-transfer path is involvement of an additional water molecule (denoted as W6) as the primary acceptor of the O6 proton. (ii) Most importantly, whereas we could include the electron transfer, coupled proton transfer and subsequent O-O bond formation in one MEP calculation, Allgöwer et al. considered energy barriers for three separate (sequential) proton transfer steps without verifying feasibility of the electron

transfer step by its direct inclusion in their calculations. (iii) Eventually Allgöwer et al. determine a high energy barrier of 16 kcal/mol for the O-O bond formation step that follows oxyl radical formation (as opposed to the computed value of about 6 kcal/mol in our study), which would imply that the slowest step in the S_3 to S_0 transition is the peroxide formation step. However, a rate-determining peroxide formation that follows fast, energetically down-hill oxyl-radical formation is in stark conflict with the experimental data on rate constants and reaction kinetics (see, e.g., ref. 10).

In summary, only the computational results of our investigation establish a direct relation to the experimental results on reaction kinetics of the oxygen-evolving S_3 to S_0 transition.

SII.7 Consideration about Proton Coupled Electron Transfer (PCET) in the slow (rate-limiting) phase of the S_3 to S_0 transition.

Our previous QM/MM MD calculations[26] suggested that the presence alone of the radical Tyr_Z is notable (in the simulation period) to trigger directly the deprotonation of O6 and the subsequent peroxide formation. However, the same MD calculations suggested that forcing the presence of a reduced Tyr_Z, and therefore its reduced hydrogen bond network around Tyr-His moiety, is enough to trigger the oxidation of O6 and proton shift from O6 to W1 in about 2 ps. This movement takes place together with Asp61 protonation, and therefore, it becomes more favorable in the case of Glu312 deprotonation, which leave the Lys317 free to drift from Asp61, lowering its pKa.

On the basis of these results and considering that there is evidence of PCET also in the previous steps of the Kok-Joliot's cycle, it is reasonable to assume that closely coupled PCET takes place also in the final transition of the catalytic cycle. To corroborate the hypothesis with additional computational results, we considered both alternatives of sequential electron and proton transfer steps:

1) the protons move before the electron transfer,
2) the electron transfer from O6 occurs without protons movements.

In the first case, we constrained the proton shared between Tyr_Z and His190 to bond the His190 nitrogen, therefore enforcing the Tyr_Z in its radical form. In this situation, the energy variation associated with the simple proton transfer leads to an increasing of the total energy of the system by +7.5 kcal/mol with respect to the starting structure. In the second case (electron transfer without proton movements), we failed to even converge a single-point electronic structure calculation, strongly suggesting that this latter hypothesis must be excluded.

In conclusion, both modes of asynchronous movement of protons and electron destabilize the system (increased total energy), whereas the closely-coupled PCET largely stabilizes it and can be considered energetically more favorable. Additionally, the necessity of large hydrogen bond network (HBN) reorganization to make the electron transfer energetically favorable corroborates the experimental evidence of a large entropic barrier for the slow phase of the transition.

SII.8 Applicability of transition state theory for the proton-coupled electron transfer step

Following Eyring's transition state theory (Eyring-Polanyi equation, [58,105] the reaction rate is related to the entropy ($\Delta S^{‡}$) and enthalpy ($\Delta H^{‡}$) of activation by:

$$k_{TST} = \frac{k_B T}{h}\, \kappa\, e^{\frac{\triangle S^{‡}}{k_B}}\, e^{\frac{-\triangle H^{‡}}{k_B T}} \qquad \text{(Eyring-Polanyi equation)}$$

and being the Boltzmann and Planck constants, respectively. The parameter κ describes the transmission probability, that is the probability that the reactions takes place once the transition state has been reached.

Based on experimentally determined rate constants and activation energies, application of above equation to the rate constant of O_2-formation results in the enthalpy of activation and entropy of activation provided in the Extended Data Figure 5 and Figure 3, for κ = 1. (We note that κ = 0.5 is a sensible choice and often used, but this difference will change the activation energies only marginally.) The thereby obtained experimental enthalpy of activation compares well to the energy-barrier value we obtained by computational chemistry, which covers not only the nuclear movements, but also the electron transfer from a substrate water molecule to the oxidized tyrosine residue. The question arises how both the application of transition state theory and the computational approach relate to electron transfer theories.

In our MEP calculations, the electron-donating Mn-cluster moiety, the electron-accepting tyrosine radical, and the surrounding protein-water network are included (Supplementary Fig. 18). Computationally, the electron transfer (ET) happens spontaneously once the nuclei have reached appropriate positions. We note that the direct coupling of the ET step to tunneling of protons through a local energetic barrier might lower the transition-state energy. Such tunneling effects are not explicitly included in the here used computational approach (and cannot be included because of too enormous computational costs). The absence of a major proton-tunneling effects is indeed in line with an experimentally observed weak H/D kinetic isotope effect of only about 1.2 for the O_2-formation step. We thus believe that the above simulation approach mirrors the experimentally observed events well. For clarity we emphasize again that the simulation approach describes a reaction proceeding by close coupling of proton relocations and electron transfer but not the simultaneous (concerted) tunneling of electrons and protons.

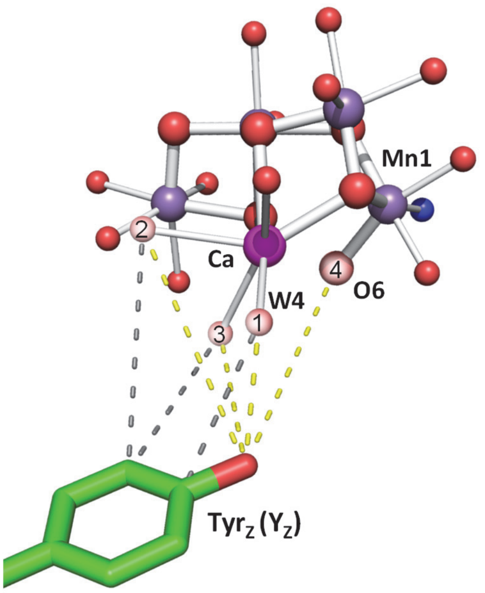

	Ligand Oxygen	Distance (Å) (Y_ZO – Lig)
1	W4	2.9
		3.7 (to CZ Ring)
2	W3	4
		4.2 (to CE1 Ring)
3	(CA1-Asp170) OD1	5.1 (to CE1 Ring)
		5.4
4	O6	5.7
-	(CA1-Ala344) O	5.7
-	(MN4-Asp170) OD2	5.8 (to CE1 Ring)
-	(MN1-Glu189) OE2	6.1
-	O1	6.2

Supplementary Fig. 21. Shortest distances between the sidechain of Tyr161 (Tyr_Z) and the first-sphere ligand atoms (all oxygen atoms) of the Mn and Ca atoms of the Mn_4Ca cluster in the S_3-state structure of ref. 25. For seven distances, connecting lines are indicated starting at four oxygen atoms, which are numbered as 1 (W4), 2 (W3), 3 (O-Asp170), or 4 (O6). The corresponding seven distances and further distances are indicated in the table.

In the framework of the Eyring-Polanyi equation, essentially instantaneous electron transfer occurs once the appropriate nuclear coordinates have been reached, which corresponds to a κ–value close to unity. In terms of non-adiabatic electron transfer theory, however, the relocation of electron density (and thus spin density) does not always occur once an appropriate nuclear geometry has been reached, but with a limited probability that is determined by the electron-tunneling distance. Following Moser et al. [106], we estimate the tunneling distance as the edge-to-edge distance between donor and acceptor moiety, where here the acceptor moiety is the tyrosine radical and the donor moiety the complex of four Mn ions and the nearest first-sphere oxygen ligands. Thereby an internuclear distance close to 5.7 Å is obtained (between Tyr161-O and O6, in the S_3 structure of ref. 25), corresponding to a Van-der-Waals tunneling distance of only about 2.5 Å. These figures predict an especially high tunneling probability. Following ref. 106, it corresponds to a free-energy optimized rate constant for electron tunneling on the order of 10^{12} s^{-1}, or a time constant of 1 ps. The frequency that describes nuclear movements in the transition state regime is about 6 times greater (k_BT/h, about $6{\cdot}10^{12}$ s^{-1} at 20°C) suggesting that indeed a minor slow-down resulting from limited ET probability cannot be excluded. Staying in the framework of transition-state theory, this slow-down by limited ET probability could described by a reduced transmission probability (reduced κ–value), which would result in a moderate reduction of the entropy of activation in the Eyring-Polanyi equation.

The above considerations involve several approximations and estimates. Possibly the tunneling probability exceeds the above estimate of 10^{12} s^{-1} because the 'medium' between the donor and acceptor moieties comprises atom groups (specifically W3 and W4) that are likely to lower the tunneling barrier significantly. Furthermore, multiple parallel tunneling paths could also increase the total tunneling probability. It is conceivable that the adiabatic limit is reached where tunneling probabilities become largely irrelevant. In conclusion, there remain uncertainties regarding the precise

numerical value of the entropy of activation, which might be moderately lower than the values in Figure 3. However, the central conclusion of a pronounced entropic slow-down will remain unaffected.

SII.9 Entropic activation-energy contribution in the slow phase of the S_3 to S_0 transition

Proposed origin of the experimentally found entropy of activation

(1) On the one hand, it is expected and verified by extensive molecular dynamics simulations on PSII that the protein internal H-bond networks (HBN) are highly dynamic, especially regarding water positions and H-bonding directionality, with a multitude of roughly isoenergetic conformations reached within nanoseconds at room temperature (see, e.g., ref. 35).

(2) On the other hand, the arrangement of the water molecules in the extended H-bonded protein-water network surrounding the Mn_4CaO_5 cluster and the Tyr_Z is well resolved in the crystallographic structures, determined at room temperature. This implies that the HBN dynamics evolve around mean positions of the individual nuclei as they are detected by protein crystallography. The HBN and the related mean-value atom coordinates are likely evolutionarily optimized for efficient (fast) water oxidation. The computational approaches used herein to obtain the starting structure of our MEP calculations favors a starting structure that is close to this evolutionary optimized HBN conformation.

In the light of (1) and (2) and our computational results on the coupling of the oxyl-radical formation step with reorganization of an extended Mn_4Ca/Tyr_Z HBN, we propose:

Although well-defined coordinates of individual oxygen atoms are resolved in crystal structures, the presence of an HBN that is at the same time and in every detail perfectly well arranged for the here discussed proton-coupled electron transfer, will still be a rare event. The limited probability to reach this perfect conformation of all atoms of the HBN explains the entropic contributions to the activation energy. The starting structures of our MEP calculations is close to this perfect conformation, which corresponds to a minimum energy conformation shaped by evolutionary optimization. Therefore, the MEP calculations can result in a meaningful enthalpy of activation, irrespective of the magnitude of the entropy of activation.

Computational prediction of the entropy of activation

An accurate and direct brute-force evaluation of the entropic contribution to the activation barrier is beyond the available computational capabilities. Entropy evaluation can be done in principle with thermodynamic integration, using multiple MD simulations covering different points of the discussed reaction coordinate. However, reaching reasonable convergence on such complex reaction coordinate could require up to several ns of QM/MM MD simulation, which is clearly beyond the currently available computational capabilities.

Nonetheless, plausible qualitative hypotheses about the entropic contribution can be developed, which may be illustrated on the grounds of insight obtained by simulations. As already discussed, a major part of the large non-enthalpic contribution can be associated with the large number of directional HB required to properly reduce the Tyr_Z by O6. In a previous work, [107] we studied the HBN stability around radical, neutral and negative Tyr_Z in water solution, reporting that, although both neutral and radical Tyr_Z are stabilized by the presence of an average of 1.5 hydrogen bonds around the oxygen atom, the oxidation is able to trigger changes in the surrounding H-bond network.

We report in Supplementary Fig. 22 (top panel) the distances of the two closest water molecules to the Tyr_Z (W4 and W5 in Supplementary Figure 18) and OH distance of the shared proton between His190 and Tyr_Z. Additionally, in the bottom panel, the distances involving the O6 hydroxide, W3 and W2 water molecules involved in the proton shuffle are reported. The distances shown in Supplementary Fig. 22 have been calculated along the QMMM-MD simulations of the S_3' and the S_4 published in ref. 26 (it should be pointed out that in the mentioned reference the S_3' and S_4 states are named differently). In the radical form (S_3', top panel), the protonated His190 interact via a stable H-bond with the Tyr_Z (purple line). Additionally, two water molecules are originally H-bonded to Tyr_Z, but before the end of the simulation the H-bond with W5 is totally lost. This is perfectly in agreement with the behavior described by Nakamura at al. [107] concerning the preferential H-bond network of a radical Tyr residue. Conversely, after reduction and re-protonation of Tyr_Z (S_4 state), the water molecule W5 comes back close to Tyr_Z forming a stable H-bond.

Being that the change of the HBN around the Tyr_Z is required to stabilize its reduced form, reached in a concerted manner with the Asp61 protonation and concerted proton shuffling between O6 and the waters W3 and W2 (see also bottom panel of Supplementary Fig. 22), an extremely wide pre-organization of an extended hydrogen bond network seems to be mandatory for Tyr_Z reduction. In our simulations such a broad, spontaneous pre-organization has never been observed due to the limited timescales typical of QM/MM simulation (ps) when compared to that associated with the transition (ms).

In summary, all the above considerations, based on several computational results of different PSII states, plausibly supports the idea that the origin of the large entropic activation-energy contribution is due to the necessity of the HBN pre-organization.

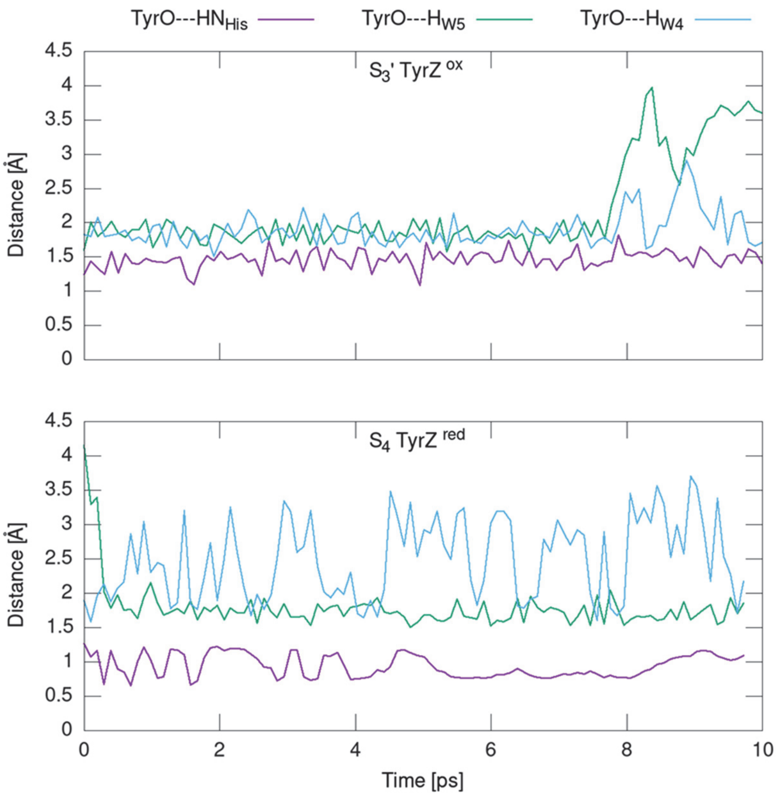

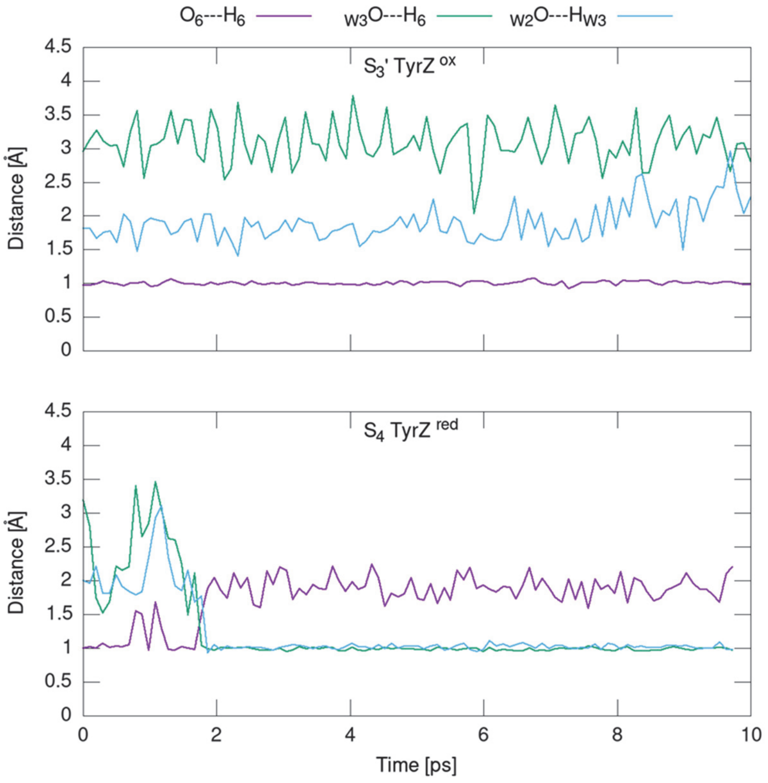

Supplementary Fig. 22. Hydrogen bond distances around Tyr$_Z$ in the S$_3$' state (top panel) and S$_4$ state (bottom panel) during QM/MM-MD simulations. The distances shown in the top panel are the O-H

distance of the shared proton between Tyr_Z and His190 (purple) and the distances between the two water molecules closest to Tyr_Z, namely W5 (green) and W4 (cyan), and the phenolic oxygen of Tyr_Z. The distances shown in the bottom panel are: between the proton of the O6-H hydroxide moiety and the W3 molecule (green); between the oxygen atom of the W2 molecule and the hydrogen of W3 (cyan); between O6 and the proton H6 bound to it in the S_4 state (cyan).

References

85 Forman, M. L., Steel, W. H. & Vanasse, G. A. Correction of Asymmetric Interferograms Obtained in Fourier Spectroscopy. *J. Opt. Soc. Am.* **56**, 59-& (1966). https://doi.org/10.1364/Josa.56.000059

86 Grabolle, M. & Dau, H. Efficiency and role of loss processes in light-driven water oxidation by PSII. *Physiol. Plant.* **131**, 50-63 (2007). https://doi.org/10.1111/j.1399-3054.2007.00941.x

87 Zaharieva, I., Grabolle, M., Chernev, P. & Dau, H. in *Photosynthesis Research for Food, Fuel and Future* (eds T. Kuang, C. Lu, & L. Zhang) 234-238 (Springer, 2013).

88 Buchta, J., Grabolle, M. & Dau, H. Photosynthetic dioxygen formation studied by time-resolved delayed fluorescence measurements--method, rationale, and results on the activation energy of dioxygen formation. *Biochim. Biophys. Acta* **1767**, 565-574 (2007). https://doi.org/10.1016/j.bbabio.2007.04.003

89 Klauss, A., Haumann, M. & Dau, H. Seven steps of alternating electron and proton transfer in photosystem II water oxidation traced by time-resolved photothermal beam deflection at improved sensitivity. *J. Phys. Chem. B* **119**, 2677-2689 (2015). https://doi.org/10.1021/jp509069p

90 Sato, A., Nakano, Y., Nakamura, S. & Noguchi, T. Rapid-Scan Time-Resolved ATR-FTIR Study on the Photoassembly of the Water-Oxidizing Mn4CaO5 Cluster in Photosystem II. *J. Phys. Chem. B* **125**, 4031-4045 (2021). https://doi.org/10.1021/acs.jpcb.1c01624

91 Menzel, J. Fourier-Transformation-Infrarot-Spektroskopie am Photosystem II zur Untersuchung des Reaktionszyklus Diploma thesis. (Freie Universität Berlin, 2009).

92 Nagao, R., Yamaguchi, M., Nakamura, S., Ueoka-Nakanishi, H. & Noguchi, T. Genetically introduced hydrogen bond interactions reveal an asymmetric charge distribution on the radical cation of the special-pair chlorophyll P680. *Journal of Biological Chemistry* **292**, 7474-7486 (2017). https://doi.org/10.1074/jbc.M117.781062

93 Okubo, T., Tomo, T., Sugiura, M. & Noguchi, T. Perturbation of the Structure of P680 and the Charge Distribution on Its Radical Cation in Isolated Reaction Center Complexes of Photosystem II as Revealed by Fourier Transform Infrared Spectroscopy. *Biochemistry* **46**, 4390-4397 (2007). https://doi.org/10.1021/bi700157n

94 Dau, H. & Haumann, M. The manganese complex of photosystem II in its reaction cycle—Basic framework and possible realization at the atomic level. *Coordination Chemistry Reviews* **252**, 273-295 (2008). https://doi.org/10.1016/j.ccr.2007.09.001

95 Chirgadze, Y. N., Fedorov, O. V. & Trushina, N. P. Estimation of amino acid residue side-chain absorption in the infrared spectra of protein solutions in heavy water. *Biopolymers* **14**, 679-694 (1975). https://doi.org/10.1002/bip.1975.360140402

96 Venyaminov, S. Y. & Kalnin, N. N. Quantitative IR spectrophotometry of peptide compounds in water (H2O) solutions. I. Spectral parameters of amino acid residue absorption bands. *Biopolymers* **30**, 1243-1257 (1990). https://doi.org/10.1002/bip.360301309

97 Senger, M. *et al.* How [FeFe]-Hydrogenase Facilitates Bidirectional Proton Transfer. *J. Am. Chem. Soc.* **141**, 17394-17403 (2019). https://doi.org/10.1021/jacs.9b09225

98 Maeda, A. *et al.* Structures of aspartic acid-96 in the L and N intermediates of bacteriorhodopsin: analysis by Fourier transform infrared spectroscopy. *Biochemistry* **31**, 4684-4690 (1992). https://doi.org/10.1021/bi00134a022

99 Takei, K., Takahashi, R. & Noguchi, T. Correlation between the hydrogen-bond structures and the C=O stretching frequencies of carboxylic acids as studied by density functional theory calculations: theoretical basis for interpretation of infrared bands of carboxylic groups in proteins. *J Phys Chem B* **112**, 6725-6731 (2008). https://doi.org/10.1021/jp801151k

100 Speakman, J. C. & Mills, H. H. 230. The crystal structures of the acid salts of some monobasic acids. Part VI. Sodium hydrogen diacetate. *Journal of the Chemical Society (Resumed)*, 1164-1175 (1961). https://doi.org/10.1039/JR9610001164

101 Li, H., Robertson, A. D. & Jensen, J. H. Very fast empirical prediction and rationalization of protein pKa values. *Proteins* **61**, 704-721 (2005). https://doi.org/10.1002/prot.20660

102 Siegbahn, P. E. M. Water oxidation mechanism in photosystem II, including oxidations, proton release pathways, O—O bond formation and O_2 release. *Biochim. Biophys. Acta* **1827**, 1003-1019 (2013). https://doi.org/10.1016/j.bbabio.2012.10.006

103 Shoji, M., Isobe, H. & Yamaguchi, K. Concerted bond switching mechanism coupled with one-electron transfer for the oxygen-oxygen bond formation in the oxygen-evolving complex of photosystem II. *Chem. Phys. Lett.* **714**, 219-226 (2019). https://doi.org/10.1016/j.cplett.2018.10.041

104 Isobe, H., Shoji, M., Suzuki, T., Shen, J.-R. & Yamaguchi, K. Exploring reaction pathways for the structural rearrangements of the Mn cluster induced by water binding in the S3 state of the oxygen evolving complex of photosystem II. *J. Photochem. Photobiol. A: Chem.* **405**, 112905 (2021). https://doi.org/10.1016/j.jphotochem.2020.112905

105 Evans, M. G. & Polanyi, M. Some applications of the transition state method to the calculation of reaction velocities, especially in solution. *J. Chem. Soc. Faraday Trans.* **31**, 875-894 (1935). https://doi.org/10.1039/TF9353100875

106 Moser, C. C., Keske, J. M., Warncke, K., Farid, R. S. & Dutton, P. L. Nature of biological electron transfer. *Nature* **355**, 796-802 (1992). https://doi.org/10.1038/355796a0

107 Nakamura, S., Capone, M., Narzi, D. & Guidoni, L. Pivotal role of the redox-active tyrosine in driving the water splitting catalyzed by photosystem II. *Phys. Chem. Chem. Phys.* **22**, 273-285 (2020). https://doi.org/10.1039/C9CP04605D
