# Supplementary material for: The electron–proton bottleneck of photosynthetic oxygen evolution
Source: Nature. 2023 May 3;617(7961):623–8. doi: 10.1038/s41586-023-06008-5 (PMC10191853; doi:10.1038/s41586-023-06008-5)
Supplement: Supplementary file 2 — Reporting Summary [file 41586_2023_6008_MOESM2_ESM.pdf]

nature portfolio

Corresponding author(s): Holger Dau

Last updated by author(s): Mar 6, 2023

Reporting Summary

Nature Portfolio wishes to improve the reproducibility of the work that we publish. This form provides structure for consistency and transparency in reporting. For further information on Nature Portfolio policies, see our Editorial Policies and the Editorial Policy Checklist.

Statistics

For all statistical analyses, confirm that the following items are present in the figure legend, table legend, main text, or Methods section.

n/a	Confirmed	
☐	☒	The exact sample size (n) for each experimental group/condition, given as a discrete number and unit of measurement
☒	☐	A statement on whether measurements were taken from distinct samples or whether the same sample was measured repeatedly
☒	☐	The statistical test(s) used AND whether they are one- or two-sided<br>*Only common tests should be described solely by name; describe more complex techniques in the Methods section.*
☒	☐	A description of all covariates tested
☒	☐	A description of any assumptions or corrections, such as tests of normality and adjustment for multiple comparisons
☐	☒	A full description of the statistical parameters including central tendency (e.g. means) or other basic estimates (e.g. regression coefficient) AND variation (e.g. standard deviation) or associated estimates of uncertainty (e.g. confidence intervals)
☒	☐	For null hypothesis testing, the test statistic (e.g. F, t, r) with confidence intervals, effect sizes, degrees of freedom and P value noted<br>*Give P values as exact values whenever suitable.*
☒	☐	For Bayesian analysis, information on the choice of priors and Markov chain Monte Carlo settings
☒	☐	For hierarchical and complex designs, identification of the appropriate level for tests and full reporting of outcomes
☒	☐	Estimates of effect sizes (e.g. Cohen's d, Pearson's r), indicating how they were calculated

Our web collection on statistics for biologists contains articles on many of the points above.

Software and code

Policy information about availability of computer code

Data collection	Only instrument-specific code was used that is irrelevant for our results and conclusions.
Data analysis	Two short Python toolboxes were used to correct time-resolved Fourier transform data of high activity Photosystem II membrane particles from spinach for starting population and cycle inefficiency (miss factor) and to perform a global fit of a data set to generate decay-associated spectra (DAS). These are made available at the Zenodo repository (doi: 10.5281/zenodo.7682034). Dependency versions: Python 3.7.9, Numpy 1.19.2, Scipy 1.5.2, Pandas 1.2.2., Matplotlib 3.3.2.

For manuscripts utilizing custom algorithms or software that are central to the research but not yet described in published literature, software must be made available to editors and reviewers. We strongly encourage code deposition in a community repository (e.g. GitHub). See the Nature Portfolio guidelines for submitting code & software for further information.

Data

Policy information about availability of data

All manuscripts must include a data availability statement. This statement should provide the following information, where applicable:

- Accession codes, unique identifiers, or web links for publicly available datasets
- A description of any restrictions on data availability
- For clinical datasets or third party data, please ensure that the statement adheres to our policy

The complete step-scan data obtained for 230.000 sequences of 10 laser flashes applied to dark-adapted PSII is available in form of averaged time courses of the detector signal for all 334 mirror positions and 10 exciting laser flashes per mirror position (deposited at Zenodo, doi: 10.5281/zenodo.7681840). We furthermore provide the spectrum used for correction of the so-called heat artefact at the same location. Using this data and the also deposited Python libraries, all steps of the data analysis can be reproduced.

The coordinates of the 14 MEP structures are provided as a Supplementary Data file in standard PDB format.

Field-specific reporting

Please select the one below that is the best fit for your research. If you are not sure, read the appropriate sections before making your selection.

☒ Life sciences ☐ Behavioural & social sciences ☐ Ecological, evolutionary & environmental sciences

For a reference copy of the document with all sections, see nature.com/documents/nr-reporting-summary-flat.pdf

Life sciences study design

All studies must disclose on these points even when the disclosure is negative.

Sample size	For signal averaging in the step-scan experiment, about 230,000 measurement cycles (230,000 sequence of 10 laser flashes) were performed and the recorded data was averaged. Further details are reported in the Methods section of the article. The number of flash sequences was chosen such that the noise in the IR time courses could not affect our conclusions.<br>In panel e of the Extended Data Fig. 5, at each temperature the data points (filled circles) indicate the average of three values of TAUox obtained by simulation of the O2-transients of three independent experiments with averaging of 70-80 flash-induced O2-transients per experiment; the error bars indicate the corresponding standard deviation (n = 3).
Data exclusions	Prior to measurement, the OD of each spot is sampled, and those not meeting the criteria of 1 +/- 0.2 OD are excluded from further measurement. This typically resulted in the exclusion of about 10% of the spots.
Replication	We tracked 230,000 excitation cycles of dark-adapted photosystems with microsecond infrared spectroscopy. The resulting data was averaged in order to obtain a single high-quality data set. Further replicates were not approached. The data quaility can be judged by visual inspection of time courses at specific wavenumbers shown in various figures of the article.<br>In panel e of the Extended Data Fig. 5, at each temperature the data points (filled circles) indicate the average of three values of TAUox obtained by simulation of the O2-transients of three independent experiments with averaging of 70-80 flash-induced O2-transients per experiment; the error bars indicate the corresponding standard deviation (n = 3).
Randomization	The topic of our study is a biological one, but the experimental biophysical approach and methodology is typical for physical sciences. It is not an empirical investigation where randomization can be applied.
Blinding	The topic of our study is a biological one, but the experimental biophysical approach and methodology is typical for physical sciences. It is not an empirical investigation where blinding can be applied.

Reporting for specific materials, systems and methods

We require information from authors about some types of materials, experimental systems and methods used in many studies. Here, indicate whether each material, system or method listed is relevant to your study. If you are not sure if a list item applies to your research, read the appropriate section before selecting a response.

Materials & experimental systems

n/a	Involved in the study	
☒	☐	Antibodies
☒	☐	Eukaryotic cell lines
☒	☐	Palaeontology and archaeology
☒	☐	Animals and other organisms
☒	☐	Human research participants
☒	☐	Clinical data
☒	☐	Dual use research of concern

Methods

n/a	Involved in the study	
☒	☐	ChIP-seq
☒	☐	Flow cytometry
☒	☐	MRI-based neuroimaging
